# Periodontitis in Patients Receiving Haemodialysis: A Scoping Review

**DOI:** 10.1155/ijod/5490199

**Published:** 2025-12-06

**Authors:** Cassie Wong, James Pham, Gedalya Lederman, Duy Doan, Jinlong Gao, Shanika Nanayakkara

**Affiliations:** ^1^ Sydney Dental School, Faculty of Medicine and Health, The University of Sydney, Sydney, New South Wales, 2006, Australia, sydney.edu.au; ^2^ Westmead Centre for Oral Health, Westmead Hospital, Institute of Dental Research, Westmead, Australia, wslhd.health.nsw.gov.au

**Keywords:** alveolar bone loss, haemodialysis, kidney failure, periodontitis, renal dialysis

## Abstract

**Introduction:**

Periodontitis is a host‐mediated inflammation associated with microbial dysbiosis which can result in loss or periodontal attachment. This is a common oral health complication in patients with kidney failure. Haemodialysis (HD) is the primary treatment modality for kidney failure. Given the increasing prevalence of kidney failure and periodontitis, understanding their associations and implications is crucial.

**Objective:**

This scoping review explores the relationship between periodontitis and HD and examines the impact of periodontal health on clinical outcomes and oral health quality of life (QoL) in patients undergoing HD.

**Methods:**

The review followed The Joanna Briggs Institute (JBI) Scoping Review Methodology and Preferred Reporting Items for Systemic and Meta‐Analyses extension for Scoping Reviews((PRISMA‐ScR). Medline, Embase, Scopus, and grey literature were searched for peer‐reviewed English‐language articles up to December 2023. Data were summarised using thematic analysis.

**Results:**

Eighty‐one articles were included, with most studies conducted in Europe and Asia. The prevalence of periodontitis in HD patients ranged from 36.27% to 99.06%. Findings highlighted associations between periodontitis and increased risks of comorbidities such as cardiovascular disease (CVD), metabolic syndrome, and other systemic illnesses.

**Conclusion:**

Periodontitis is highly prevalent in patients on HD and associated with adverse systemic and oral health outcomes. Limitation in current evidence are heterogeneity in case definitions and lack of longitudinal studies to provide causal inference. The review recommends standardised diagnostic criteria and longitudinal studies to guide integrated care approaches.

## 1. Introduction

Chronic kidney disease (CKD) is a progressive condition marked by declining renal function, potentially advancing to end‐stage renal disease (ESRD) requiring renal replacement therapy [1]. Prevalence of renal failure is increasing worldwide, and dialysis is the predominant treatment for improving long term survival [2]. Haemodialysis (HD) is the most commonly utilised method of dialysis in patients with renal failure and used by 82% of dialysis patients in Australia in 2021 [3]. While HD prolongs survival, it is associated with increased morbidity, mortality and a reduced quality of life (QoL) compared to the general population [4, 5].

Periodontitis is a chronic host‐mediated inflammatory associated with microbial dysbiosis which can result in loss of periodontal attachment [6]. It is a major public health concern, particularly in older adults and affects over a billion people globally [7, 8]. Periodontitis can lead to significant oral complications, including deep periodontal pockets, alveolar bone resorption, tooth mobility and eventual tooth loss, which may severely impact mastication, speech and overall QoL [9]. Importantly, periodontitis is one of the most common diseases worldwide with reported increasing global prevalence [10]. Interestingly, existing evidence reports an association between periodontitis and multiple systemic diseases such as cardiovascular disease (CVD), diabetes, hypertension and CKD [11].

Increasing evidence reports that patients with renal failure, particularly those undergoing HD have higher prevalence of periodontitis recognising this as a common oral health problem in this population [12, 13]. This may be due to multiple factors such as immune system suppression, persistent systemic inflammation and pathophysiological changes in the oral environment, including altered salivary flow and composition [14, 15].

Oral care in patients undergoing HD is often suboptimal influenced by poor oral hygiene, restricted diets, medications side effects and the increasing likelihood of malnutrition [16–18]. Thus, it is important to incorporate this in the overall clinical management of these patients. Furthermore, poor periodontal health may not be limited to oral consequences. Previous studies have reported that microorganisms involved in periodontitis may spread into the bloodstream and lead to impairment of systemic markers of inflammation such as C‐reactive protein (CRP), potentially contributing to systemic consequences including CVDs and mortality in patients with renal failure [19, 20]. Periodontitis has also been suggested to be a risk factor for progression of renal impairment and is associated with increased mortality in patients undergoing long term HD [20]. These studies suggest that the clinical outcomes of these patients have a direct association with their periodontal health.

With an ageing population, a growing number of individuals requiring dialysis and the high prevalence of periodontitis in patients receiving HD, it is important to understand the association between periodontitis and HD that would help in developing evidence‐based recommendations to improve clinical outcomes of HD patients. Despite a growing amount of research highlighting the relationship between periodontitis, renal failure and dialysis, there has been no recent reviews on the association between HD and periodontitis. Thus, this scoping review was conducted to explore the extent of knowledge, on the association between periodontitis and HD in patients with ESRD. The specific objectives were to (i) report the prevalence of periodontitis among HD patients, (ii) identify associated oral and systemic complications, (iii) evaluate the oral health‐related QoL (OHRQoL) in HD patients with periodontitis and (iv) to examine the role of periodontal treatment on clinical outcomes of HD patients.

## 2. Methods

### 2.1. Search Strategy

This scoping review was conducted in accordance with Joanna Briggs Institute (JBI) Scoping Review Methodology and reported based on the Preferred Reporting Items for Systemic and Meta‐Analyses

extension for Scoping Reviews(PRISMA‐ScR) [21]. Medline, Embase and Scopus databases were searched systematically to identify relevant literature, published in English language up to December 2023. A grey literature search was also conducted to supplement the database search. Primary research conducted on humans including both interventional (e.g., clinical trials) and observational studies (e.g., cohort studies and case–control studies) were selected. Any in‐vitro studies, animal studies, review articles and editorials were excluded. To ensure a comprehensive coverage of the broad literature, no data restrictions were applied. MeSH terms and keywords including periodontal disease, periodontitis, periodontal health, alveolar bone loss, dialysis, HD, renal dialysis were used with appropriate wildcards and Boolean operators (Figure S1).

### 2.2. Selection Process

**Figure 1:**
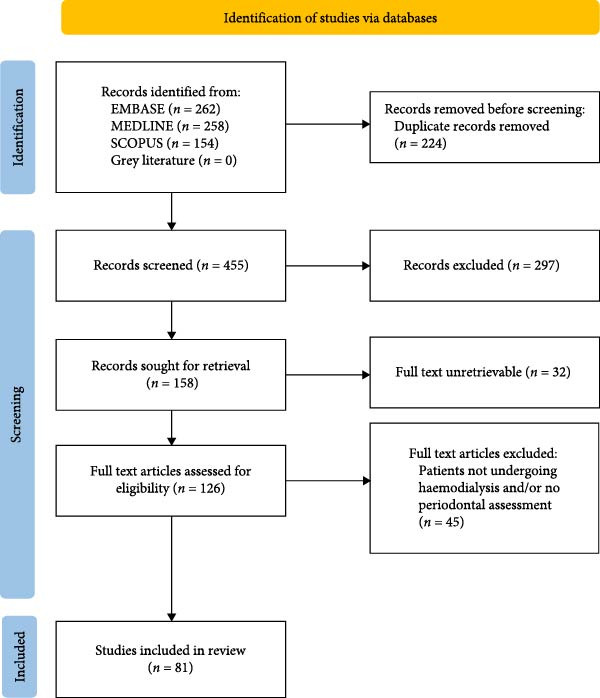
Article selection process [22].

Records identified through database searching were imported into EndNote reference management software and duplicates removed. Titles and abstracts of the selected articles were independently screened for relevance to the research aims by two reviewers. Selected full texts were reviewed independently and disagreements were resolved with two additional reviewers. Studies were included based on the inclusion criteria: (a) Patients with renal failure on HD and (b) studies with reported periodontitis or periodontal parameters. Exclusion criteria include: (a) Patients not on HD,(b) patients on HD not as a result of chronic renal failure, for example, acute kidney injury and (c) no periodontal health parameters reported. The results of the search and study selection process is presented in the PRISMA flowchart (Figure 1).

### 2.3. Data Extraction and Analysis

Data from the selected articles was extracted by four independent reviewers using an Excel spreadsheet. The data extracted from the selected articles include name of the first author, year of publication, country of origin, study design, participant characteristics, renal and periodontal parameters. Any disagreements were resolved through discussion. Descriptive summaries of article information were calculated using Google Sheets. A thematic analysis was conducted, and the data was summarised under five identified themes.

## 3. Results

### 3.1. Descriptive Summary of Studies

The article selection process resulted in 81 studies that were included in this review (Figure 1, Table S1). As illustrated in Figure 2A, studies were primarily conducted in Asia (29.6%) and Europe (23.5%), with fewer from the Middle East (22.2%), South America (14.8%), North America (4.9%) and Africa (2.5%) or multi‐region (2.5%). The most common study design used was cross‐sectional (69%), followed by case–control (13.6%), cohort (11.1%), and clinical trials (6.2%) (Figure 2B).

Figure 2Distribution of studies: (A) by location; (B) by study design.

**Figure:**
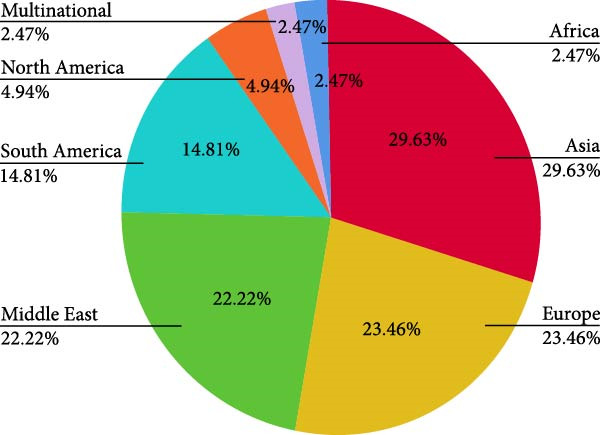
(A)

**Figure:**
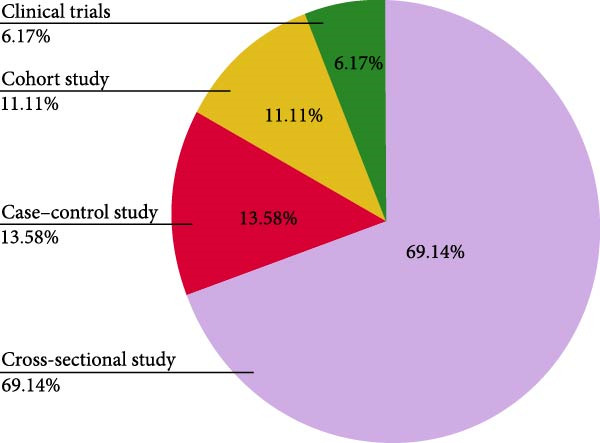
(B)

**Figure 3:**
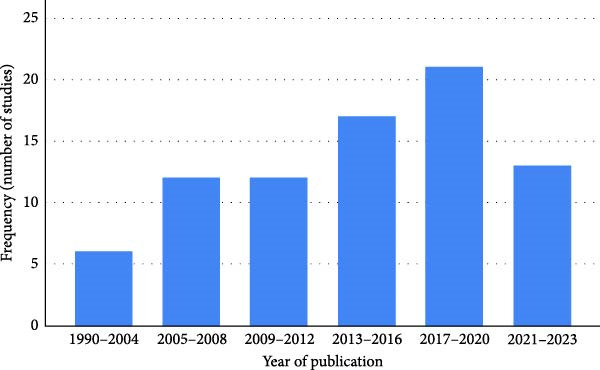
Distribution by publication year.

The analysis based on the publication year demonstrated that the majority have been published between 2013 and 2023, with a concentration between 2017 and 2020 (Figure 3).

### 3.2. Prevalence and Severity of Periodontitis

Reported prevalence rates of periodontitis in patients undergoing HD show significant variability ranging from 36.27% to 99.06% (Table S2) [23–37]. It is worth noting that these studies have used different case definitions to diagnose periodontitis as presented in Table S1. Therefore, the significant variation in prevalence reported can be attributed to different forms of periodontal measurements and classifications. The prevalence of periodontitis was also reported in relation to the age of the patients. One study reported a low prevalence of periodontitis among patients aged 30–39 and with a higher prevalence of 88.9% in patients aged 70–79 [38]. This age‐related trend was also reflected in another study, which reported had a lower Community Periodontal Index treatment needs (CPITN) score in younger subjects aged 16–19 compared to older subjects aged ≥45 [39].

Studies have reported increased severity of periodontal disease amongst patients on HD compared to healthy controls based on different clinical measurements such as probing pocket depth (PPD), attachment loss, gingival bleeding, bone loss and periodontal inflamed surface area [40–44]. For example, a study conducted among a Chinese population reported that patients receiving HD exhibited a 2.5‐fold higher number of sextants with PPD ≥4 mm, while the prevalence of clinical attachment loss (CAL) ≥6 mm was fourfold higher compared to healthy controls [45]. Another study conducted in Poland demonstrated similar results reporting high severity of periodontitis among HD patients compared to the general population [25].

However, some studies have reported no significant association between HD and periodontitis, reporting no observable differences in periodontal measurements between ESRD patients on HD compared to patients not on HD [46, 47]. Interestingly, several studies have also reported a positive correlation between the duration of HD and severity of periodontitis [27, 48–51], while conflicting evidence has been reported in other studies [52–54].

### 3.3. Oral Complications

Individuals undergoing HD with periodontitis frequently present with other oral complications such as xerostomia, higher plaque and calculus accumulation, and dental caries (Table S3) [55–60]. A large multinational, prospective study found 40% of HD patients with moderate‐to‐severe periodontitis had a high average DMFT (decayed, missing and filled tooth) score, dental erosion, dry mouth and oral pain [23]. Other complications observed were oral thrush, precancerous mucosal leukoplakia, erythroplakia lesions and oral herpes [42].

Additionally, microbial differences including elevated red complex bacteria and salivary pH with reduction of salivary flow rates were observed [23, 61, 62]. Additionally, higher levels of pro‐inflammatory cytokines such as TNF‐α and IL‐8 in the gingival crevicular fluid (GCF) have also been reported in HD patients compared to healthy controls [63]. This study also reported a strong positive correlation between the levels of these inflammatory cytokines and clinical periodontal measurements in HD patients [63]. It is important to report that this review also came across a study reporting absence of any significant differences in inflammatory cytokine levels between CKD patients receiving and not receiving HD [46]. It is worth noting that, another study demonstrated a higher prevalence of herpes simplex virus type 1 (HSV‐1) in saliva and GCF in HD patients compared to healthy patients [42].

### 3.4. Comorbidities and Mortality

Studies have reported associations between periodontal health and other systemic health conditions in HD patients, leading to adverse outcomes including increased mortality risks (Table S4) [64–66]. Higher risk of all‐cause mortality in HD patients with moderate‐to‐severe periodontitis has been reported in some studies, while some studies have not observed similar associations [67, 68]. Interestingly, a study has reported that HD patients with moderate‐to‐severe periodontitis have exhibited 5.3 times higher risk of CVD mortality compared to those with mild periodontitis or healthy periodontium [69]. In addition, diabetes has also been reported to be associated with worsening periodontal health in patients [70]. Further, a 2.736‐fold higher risk of metabolic syndrome in HD patients with moderate‐to‐severe periodontitis compared to healthy controls has also been observed [71, 72]. Multiple studies have also observed signs of systemic inflammation in HD patients with periodontitis, with elevated levels of CRP, amongst other inflammatory biomarkers such as IL‐6 and IL‐8 [46, 63, 73, 74].

HD patients with severe periodontitis were 2.64 times more likely to exhibit elevated malnutrition–inflammation‐atherosclerosis (MIA) components [75] and had lower serum albumin levels [76–78] compared to those without severe disease. Kovacevic et al. reported poor nutritional status, indicated by higher subjective global assessment (SGA) scores, was associated with increased mortality, with each one‐unit increase corresponding to a 62% higher risk of death [79]. In contrast, a study on the association between sarcopenia and periodontitis in HD patients have not demonstrated similar association [80].

### 3.5. OHRQoL

Periodontitis can lead to complications such as alveolar bone resorption, tooth mobility and tooth loss which may severely impact mastication, speech, and individuals’ OHRQoL (Table S5) [9]. Studies have reported high prevalence of edentulism and overall poor oral hygiene among HD patients [58]. Additionally, HD patients had demonstrated significantly lower scores on the Geriatric Oral Health Assessment Index (GOHAI‐1) with individuals missing two or more anterior occluding pairs often scoring under 40 (25th percentile [81]). Further, multiple studies have reported poorer oral hygiene and lower QoL scores among HD patients with severe periodontitis [31, 32, 82]. Studies showed that HD patients with periodontitis had reduced scores on the SF‐36 health survey along with elevated periodontal parameters such as probing depth (PD), gingival index (GI) and plaque index scores [26, 83, 84]. Similar findings were observed using the Oral Health Impact Profile (OHIP‐14), which also demonstrated lower scores among HD patients compared to healthy controls [26]. Importantly, a study has reported that HD patients with periodontitis perceive oral health care as a lower priority relative to the systemic health challenges associated with HD [53]. It is worth noting that psychological comorbidities such as depression and anxiety have also been reported frequently in this population [26].

### 3.6. Effects of Periodontal Treatment

Periodontal treatment in HD patients has demonstrated reductions in systemic inflammatory markers. Multiple studies have reported decreases in serum CRP levels, as well as improvements in blood urea nitrogen and serum albumin, following periodontal therapy (Table S6) [77, 85–88]. However, few studies have also reported that there has been no significant impact of periodontal treatment on mortality, albumin or creatinine levels [89, 90]. Periodontal treatment was also found to improve estimated glomerular filtration rates (eGFR) in HD patients and also a notable decrease in asymmetric dimethylarginine (ADMA) levels, a marker of endothelial function [91]. In addition, studies have also demonstrated that intensive periodontal treatment can reduce the overall risk of infectious diseases, including acute and subacute infective endocarditis, pneumonia and osteomyelitis, in HD patients [13]. Despite this, studies have highlighted that maintaining oral health and managing periodontitis remains challenging for individuals undergoing HD [44, 92].

## 4. Discussion

This scoping review identified a high prevalence of periodontitis among patients undergoing HD, with most studies reporting moderate‐to‐severe periodontitis. Existing evidence demonstrates that individuals undergoing HD are also at an increased risk of various oral complications including xerostomia and dental caries. In addition, studies have demonstrated systemic complications and increased mortality associated with periodontitis in HD patients as well as OHRQoL. The presence of these oral health issues highlights the need for tailored oral care strategies to mitigate the adverse effects on overall health and QoL. Further, the positive health effects of periodontal treatment on HD patients suggests a potential avenue for managing both oral and systemic health outcomes in HD patients. However, the current evidence is not sufficient to draw causal inferences or recommend evidence‐based clinical recommendations due to the conflicting and poor‐quality evidence and significant heterogeneity of periodontitis case definitions in the included studies.

Age is a well‐established risk factor for both CKD and periodontitis with the prevalence of both conditions increasing significantly with advancing years [93, 94]. Previous studies suggest that the bidirectional relationship between CKD and periodontitis may be more pronounced in older individuals, as CKD‐related systemic inflammation and immune dysregulation can accelerate periodontal destruction, while chronic periodontal infection further contributes to systemic inflammation and renal decline [95]. The higher prevalence of periodontitis observed among older HD patients compared with their younger counterparts may reflect this association. Periodontitis in HD patients is also associated with other adverse outcomes, including increased mortality risks, CVD mortality, metabolic syndrome and pneumonia‐related mortality which are also associated with advanced age [66, 67, 69, 71]. Existing evidence though, is conflicting, suggests the benefits of integrated oral care strategies in HD patient management to mitigate the burden of comorbidities, reduce the mortality risk and improve overall patient outcomes including OHRQoL [77, 85–88]. However, managing oral health can be complicated in these patients due to various reasons such as comorbidities, age‐related oral health problems, patients’ attitudes and perception towards oral hygiene [71, 81].

The heterogeneity in case definitions of periodontitis was a major limitation across the literature. Many studies did not specify diagnostic criteria or used inconsistent thresholds for PPD or CAL. This inconsistency hampers comparability across studies and may contribute to the wide variation in reported prevalence. Future research should adopt up to date standardised classifications such as the 2017 Classification of Periodontal and Peri‐Implant Diseases and Conditions to enable better synthesis of findings [96]. Lack of consideration or adjustments for confounders including numerous comorbidities due to overall systemic issues in these HD patients with ESRD also negatively impacts the quality of evidence reported int these studies. The predominance of cross‐sectional and observational studies limits the ability to draw causal conclusions and evidence‐based clinical recommendations. Finally, a notable gap exists in the geographic distribution of research with absence of studies originated from Oceania, despite the high burden of CKD in countries such as Australia and New Zealand [96]. Future studies should also prioritise diverse populations such as the indigenous populations who experience disproportionate rates of both CKD and periodontitis [96, 97].

## 5. Conclusion

The review demonstrates a high prevalence of periodontitis amongst patients with ESRD receiving HD and its association with comorbid conditions, OHRQoL and mortality. Evidence, though limited, suggests that periodontal treatment in HD patients may help reduce inflammatory markers and improve biological parameters. While an interdisciplinary approach may help in reducing complications and improve outcomes, inconsistencies in case definition of periodontitis in current literature and lack of clinical trials and longitudinal studies along with conflicting evidence highlights the need of well‐designed prospective studies using standardised periodontal assessment protocols to improve the understanding on causal relationship periodontitis and HD, as well as the underlying biological mechanisms.

## Ethics Statement

The authors have nothing to report.

## Conflicts of Interest

The authors declare no conflicts of interest.

## Author Contributions

Cassie Wong, James Pham, Gedalya Lederman, Duy Doan and Shanika Nanayakkara contributed to the conceptualisation of this review. Cassie Wong, James Pham, Gedalya Lederman and Duy Doan contributed to the acquisition, analysis, and interpretation of data and drafted the manuscript. Jinlong Gao and Shanika Nanayakkara critically revised the manuscript. Shanika Nanayakkara provided supervision and mentorship.

## Funding

This work has not received support from any funding scheme.

## Supporting Information

Additional supporting information can be found online in the Supporting Information section.

## Supporting information


**Supporting Information** Figure S1: Medline search strategy. Table S1: Summary of the articles included in the review. This supporting table provides a summary of all articles included in this review. Table S2: Prevalence and severity of periodontitis. Table S3: Oral complications. Table S4: Comorbidities and mortality. Table S5: Oral health related quality of life (OHRQoL). Table S6: Effects of periodontal treatment.

## Data Availability

The data that supports the findings of this study are available in the supporting information of this article.
