# Supplementary material for: Periodontitis in Patients Receiving Haemodialysis: A Scoping Review
Source: Int J Dent. 2025 Dec 6;2025:5490199. doi: 10.1155/ijod/5490199 (PMC12752909; doi:10.1155/ijod/5490199)
Supplement: Supplementary file 1 — Supporting Information Figure S1: Medline search strategy. Table S1: Summary of the articles included in the review. This supporting table provides a summary of all articles included in this review. Table S2: Prevalence and severity of periodontitis. Table S3: Oral complications. Table S4: Comorbidities and mortality. Table S5: Oral health related quality of life (OHRQoL). Table S6: Effects of periodontal treatment. [file IJOD-2025-5490199-s001.docx]

**Supplementary Figure 1: Medline Search Strategy**

*
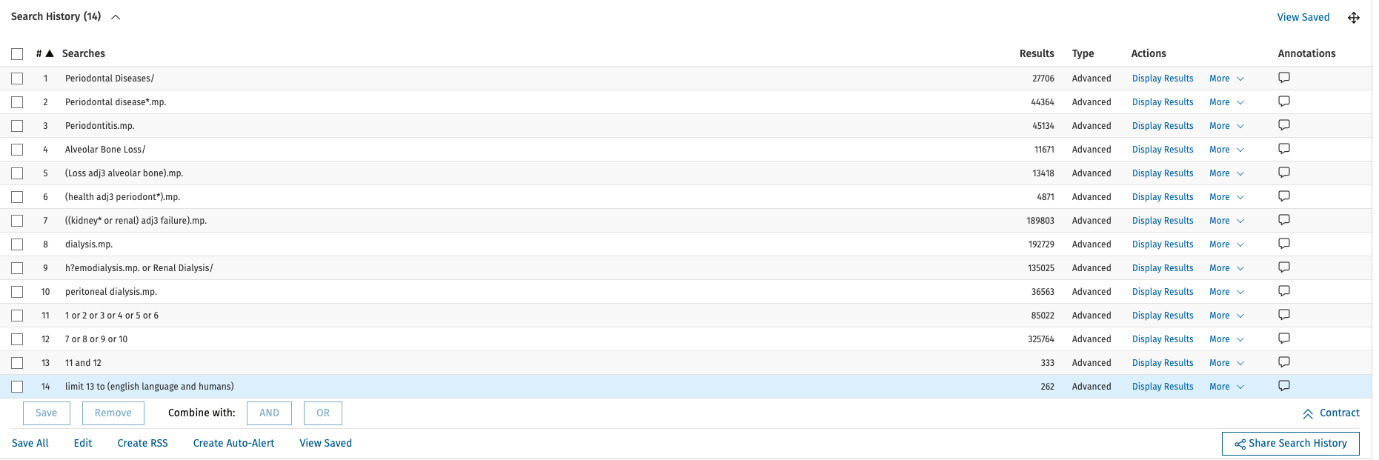
*

**Supplementary Table 1. Summary of the articles included in the review**

| **First Author (year of publication)** | **Region (Country)** | **Study Design** | **Data Collection Methods** | | **Target population** | **Study size (gender)** | **Mean Age (years)** | **Mean dialysis time** | **Periodontal Definitions** |
| --- | --- | --- | --- | --- | --- | --- | --- | --- | --- |
| Gavaldá.C (1999) | Europe (Spain, UK) | Case control study | | Clinical Examination | HD patients vs healthy controls | n= 185; HD: 53 M and 52 F, Healthy: 29 M and 24 F | HD: 58.9±14.9 years; Healthy: 55.76±10.7 | 59.8±43.9 months. | Periodontal health determined by hygiene indices and loss of periodontal attachment |
| Amara Swapna. L (2013) | Asia (India) | Case control study | | Clinical Examination | Diabetic and non diabetic patients on HD | Nondiabetic = 50, Diabetic = 47 | non-diabetic: 55.34±10.56; diabetic: 53.78±11.77 | non-diabetic: 3.98±1.79 years;  diabetic: 3.13±.1.41 years | CPITN index |
| Jenabian.N (2013) | Middle east (Iran) | Cross sectional study | | Clinical Examination | HD patients | n = 115; 63 M and 52 F | 47.9±15.3 years | N/A | Periodontal parameters including PI (PI), gingival index (GI), CAL (CAL) and PPD (PPD) |
| Ma.L (2016) | Asia (Bejing, China) | Cross sectional study | | Multiple: Clinical examination, patient interview | HD patients and PD patients, healthy controls | HD = 49 (M = 62.5%) PD = 38 (M = 60.5%) Control = 40 (M = 46.2%) | Control group: 54.38±11.96;   HD group: 54.45±15.34;  PD group: 59.29±15.00 | N/A | N/A |
| Almeida. S (2017) | South America (Brazil) | Cohort study | | Multiple: Clinical Examination, biochemical testing | HD patients | n = 26; 13 M | 59.8 ± 12 years | 3.4±2.3 years | Generalized chronic periodontitis: ≥ 30% PPD >4  Severe periodontitis: ≥ 2 sites with CAL >6mm and PPD > 5mm |
| Veisa.G (2017) | Europe (Romania) | Cross sectional study | | Multiple: Clinical examination, patient interview | HD patients | n = 101; 57 F | 52.5 ±14.3 | 6.7±5.6 years | Gingival and Periodontal Index > 3 |
| Parente.I.T (2018) | South America (Brazil) | Case control study | | Multiple: Clinical examination, patient interview | HD patients | HD = 45 (68.8% M) Healthy controls = 26 (42.3% M) | HD: 48.59 ± 17.73 Healthy: 37.88 ± 14.21 | N/A | N/A - individual parameters were compared in study vs control groups |
| Noor, H (2023) | Asia (Pakistan) | Cross sectional study | | Multiple: Clinical examination, patient interview | CKD Stage 3, 4, 5 Age: ≥ 18 Exclusion: Dialysis for reasons other than CRF, systemic illness | n = 32 (55.6% M) | 33.08 ± 17.042 | N/A | N/A |
| Rahman. M.M (1992) | Middle East (Turkey) | Cross sectional study | | clinical Examination | HD patients and patients with renal transplant | Renal transplant = 54 (45 M, 9 F) CRF = 52 (41 M, 11 F) | 32.25 | N/A | N/A |
| Frankenthal. SF (2002) | Middle East (Israel) | Case control study | | Clinical Examination | E Group: HD patients with Secondary Hyperparathyroidism (HPT) and CRF  C Group: Healthy Controls | E group: 35  C group: 35 | 33 | N/A | Alveolar bone loss |
| Rahmati. MA (2002) | North America | Cross sectional study | | Biochemical testing | HD patients | n = 86 (46 M) | 53.3 ± 16.1 | 22 months (range, 0 to 148 months) | N/A |
| Marakoglu.I (2003) | Middle East (Turkey) | Cross sectional study | | Clinical Examination | HD patients and healthy controls | HD = 36 (20 M)  Controls = 36 (20 M) | HD: 50.4±14.2 Control: 50.2±12.4 | <1 year 1-3 years >3 years | 0: Absence of inflammation 1: Mild to moderate inflammation - papilla only 2: Mild to moderately severe gingivitis 3: Severe gingivitis characterized by; redness, swelling, bleeding and ulceration 4: ≥2 pockets of ≤3mm from the CEJ 5: ≥2 pockets of 3-6mm from the CEJ 6: ≥2 pockets of ≥6mm from the CEJ |
| Duran. I (2004) | Middle East (Turkey) | Cross sectional study | | Clinical Examination | HD patients and PD patients | 16–19 = 17 (9 M)  20–34 = 92 (51 M)  35–44= 62 (35 M)  ≥45 =171 (84 M) | 43.7±15.5 (range 16-74 years) | N/A | N/A |
| Chen. LP (2006) | Asia (Taiwan) | Cross sectional study | | Multiple: Clinical examination, biochemical testing | HD Patients | HD = 253 (53.8% F) | 58.8 ± 0.8 | 48.6 ± 3.2 months | No periodontitis: PDI 0-3  Mild periodontitis: PDI 3-4   Moderate periodontitis: PDI 4-5  Severe periodontitis: PDI 5-6 |
| Kadiroglu. AK (2006) | Middle East (Turkey) | Cross sectional study | | Clinical examination Biochemical testing | HD Patients.  Group 1 = elevated CRP Group 2 = normal CRP | Group 1 = 21 (12 F)  Group 2 = 20 (10 F) | 43.1±13.1 | Group 1: 23.4±23.4 months  Group 2: 26.4±25.2 months.   Patients were on regular 5-hour maintenance HD program 3 times/week | 0: Absence of inflammation 1: Mild to moderate inflammation - papilla only 2: Mild to moderately severe gingivitis 3: Severe gingivitis characterized by; redness, swelling, bleeding and ulceration 4: ≥2 pockets of ≤3mm from the CEJ 5: ≥2 pockets of 3-6mm from the CEJ 6: ≥2 pockets of ≥6mm from the CEJ |
| Bayraktar. G (2007) | Middle East (Turkey) | Cross sectional study | | Clinical Examination | HD Duration < 3 years: n=17 HD Duration > 3 years: n=59 | HD = 76 (36 M) Healthy Controls = 61 (22 M) | HD: 48±15  Control: 46±18 | N/A | N/A |
| Borawski. J (2007) | Europe (Poland) | Cross sectional study | | Clinical examination Clinical record analysis Patient interview | HD Patients, CAPD Patients, Pre-Dialysis Patients  vs  Healthy Controls with advanced periodontitis, Healthy Controls | HD = 35 (31% F) CAPD = 33 (42% F) Pre-Dialysis = 38 (53% F)  Healthy Controls - advanced perio = 26 (38% F)  Healthy Controls = 30 (50% F) | HD: 56±11 CAPD: 51±11 Pre-Dialysis: 51±15 Healthy Controls with advanced periodontitis 49±7 Healthy Controls: 47±10 | HD: 14months (2-206) CAPD: 14months (3-72) | N/A |
| Castillo. A (2007) | Europe (Spain) | Cross sectional study | | Multiple: clinical examination, biochemical testing, PCr | HD Patients Healthy Controls | HD = 52 (24 M)  Controls =52 (24 M) | HD: 61.5 Control: 59.8 | 43.4±38.96 months | % of sites with CAL ≥3mm: 0% = absent, 0–32% = mild, 33–66% = moderate and 67–100% = severe |
| Cunha. FL (2007) | South America (Brazil) | Cross sectional study | | Multiple: Clinical examination, clinical record analysis, patient interview | CRF patients undergoing HD | HD = 160 (91 M) | 59±12 (range = 40-85) | 24 (11months-11years) | N/A |
| de Souza. CM (2007) | South America (Brazil) | Cross sectional study | | Multiple: Clinical examination, PCR, biochemical testing, | Group 1 = CKD without periodontitis, Group 2 = no CKD with periodontitis, Group 3 = ESRD on HD without periodontitis, Group 4 = ESRD on HD with periodontitis | Group 1 = 59, Group 2 = 50, Group 3 = 50, Group 4 = 63 | 44.9 (range 23–77) | Without Periodontitis = 47.8,  With Periodontitis = 47.2 | Based on clinical parameters: PPD, CAL at 4 points around each tooth. CAL > 5mm, in at least 3 teeth in at least 2 quads were considered affected with PD |
| Kshirsagar. AV (2007) | North America (United States) | Cross sectional study | | Multiple: Clinical examination, biochemical testing | HD patients | Total 154 (85M,69F) Cases: 35 (20M,15F) Non-cases: 119 (58M,61F) | Total: 54.6±13.3 Cases: 62.3±12.1 Non-cases: 52.2±12.7 | Total: 4.0±3.2 years Cases: 4.0±3.3 years Non-cases: 3.9±3.2 years | Periodontitis: ≥60% of sites with AL ≥4 mm |
| Takeuchi. Y (2007) | Asia (Japan) | Case control study | | Multiple: clinical examination, PCR, biochemical testing, | patients with renal disease (non-haemodialysis, haemodialysis) and healthy controls | Total 143 Diseased: 81  Control: 62  No gender breakdown | Diseased: 61.6 ± 9.84 Control: 57.6 ± 15.8 | 10.24 ± 8.10 years | Four sites with PPD >4mm |
| Baioni. CS (2008) | South America (Brazil) | Case control study | | Multiple: clinical examination, pcr | Group 1, healthy patients;   Group 2, without CKD and with periodontal disease;   Group 3, with CKD and without periodontal disease;   Group 4, with CKD and periodontal disease | Total n=224 1: 60 (43F,17M)  2: 50 (33F/17M)  3: 50 (16F/34M)   4: 64 (23F/41M) | 1: 37.8±9.6   2: 40.8±9.4   3: 45.2±12.9   4: 54.5±12.2 | 3: 47.8 ± 48.0 months   4: 47.2 ± 43.4 months | Periodontitis: CAL≥5 mm, in at least three teeth, in at least two quadrants |
| Bayraktar. G (2008) | Middle East (Turkey) | Cross sectional study | | Clinical Examination | Patients on HD and PD and healthy controls | PD patients: 75 (n=32 M)  HD patients: 41 (n=23 M)   C: 61 (n=22 M) | PD = 44 ±12 HD = 46 ± 15) Healthy Controls = 46 ± 18 | N/A | N/A |
| Cengiz. MI (2009) | Middle East (Turkey) | Case Control Study | | Clinical Examination | HD Patients Healthy Controls | Total n=109 HD: 68 (37M/31F) Control: 41 (21M/20F) | HD:47.85 ± 14.61  Control: 44.80 ± 10.22 | 11 patients <1 year  13 patients 1–2.9 years  18 patients 3–4.9 years  18 patients 5–9.9 years  8 patients >10 years  HD patients undergoing 4h of HD thrice a week. | N/A |
| Guzeldemir. E (2009) | Middle East (Turkey) | Cross sectional study | | Multiple: clinical examination, patient interview, biochemical testing | HD patients | HD = 47 (23 F, 24 M) | mean age, 46.38 ± 15.10 years | 71.04 ± 1.16 months | N/A |
| Kshirsagar. AK (2009) | North America (United States) | Cohort study (Retrospective) | | Multiple: Clinical examination, biochemical testing | HD patients | HD = 168 No/Mild Perio =100 (54M/46F) Moderate/Severe = 64 (23M/41F) | no/mild: 52.2 ± 13.0 Mod/Sev: 55.7 ±13.3 | No/Mild: 3.8 ± 2.7 years Mod/Sev: 4.0 ± 3.8 years | Periodontal disease: presence of significant attachment (level) loss and pocket depth. Moderate-to-severe periodontitis: 2 or more teeth with at least 6 mm interproximal attachment level and at least 1 site with probing depth >5 mm. Mild or no periodontitis: absence of findings |
| Thorman. R (2009) | Europe (Sweden) | Cross sectional study | | Clinical Examination | Predialysis, PD and HD | Overall: 62 M (66%) 31 F (34%)  Predialysis =68 PD =19 HD =15 | 61 (30-89) | N/A | N/A |
| Dağ. A (2010) | Middle East (Turkey) | Cross sectional study | | Clinical examination Biochemical testing | HD patients, Healthy controls | Total: 86 HD: 43 (20M/23F) Healthy control: 43 (21M/22F) | HD: 42.02±17.05 Control: 41.11 ± 16.13 | 36.0±1.70 months | PPD was used to identify periodontal disease. Patients were then grouped as having periodontitis or gingivitis, according to their pocket depth |
| Torres. SA (2010) | South America (Brazil) | Cross sectional study | | Clinical examination Biochemical testing | HD patients with CRF and Healthy Controls with periodontitis | Total n=30 CRF: n=16 (12M/4F) Control: n=14 (10M/4F) | CRF: 41.7±7.2  Control: 41.4±7.6 | 29.1±22.4 months months | BANA test is a chair-side in vitro test, can detect the presence of one or more anaerobic bacteria associated with periodontal disease in subgingival dental plaque samples |
| Chen. LP (2011) | Asia (Taiwan) | Cohort study (Prospective) | | Clinical examination Clinical record analysis | HD Patients | HD patients: n=253 (136F) | 58.8 ± 0.8 | 48.8 ± 3.2 months | No/Mild periodontitis: PDI 0-4 Moderate periodontitis: PDI 4-5 Severe periodontitis: PDI 5-6 |
| Chen L.P (2011) | Asia (Taiwan) | Cross sectional study | | Clinical examination Clinical record analysis | HD Patients | n=253 (136F,117M) | 58.8 ± 0.8 | 48.8 ± 3.2 months | No/Mild periodontitis: PDI 0-4 Moderate periodontitis: PDI 4-5 Severe periodontitis: PDI 5-6 |
| Brito. F (2012) | South America (Brazil) | Cross sectional study | | Clinical examination Patient interview | CAPD Patients HD Patients Predialysis Patients Healthy Controls | Total n=198 CAPD patients: n=40 (10M/30F) HD patients: n=40 (21M/19F) Predialysis patients: n=51 (29M/22F)  Healthy Controls: n=67 (23M/44F) | CAPD patients 52±12,  HD patients 50±10,  Predialysis patients 54±11  Healthy Controls 50±7 | 73.4 ± 56.2 months | Severe chronic periodontitis: ≥4 sites with CAL ≥ 6 mm and at least 30% of sites with CAL ≥ 4 mm  Generalized chronic periodontitis: ≥30% of sites with CAL ≥ 4 mm |
| Sekiguchi. RT (2012) | South America (Brazil) | Cross sectional study | | Clinical Examination | Group L: HD ≤ 36 months Group M: HD > 37 months | Group L: 50 (n=26 M)  Group M: 44 (n=25 M) | Group L: 20-39 yrs: n=28 40-79 yrs: n=22  Group M:  20-39 yrs: n=16 40-79 yrs: n=28 | 1-5 years | N/A |
| Siribamrungwong. M (2012) | Asia (Thailand) | Clinical Trial | | Clinical Examination | HD Patients | HD patients: n=30 (18M/12F) | 62 ± 15 | N/A | PDI scores: 0: no inflammation 1: mild gingivitis 2: moderate gingivitis 3: advanced gingivitis 4, ≤3 mm CAL 5, 3 to 6 mm CAL 6,: >6 mm CAL The PDI score of 0–3 is defined as no periodontitis |
| Teratani. G (2013) | Asia (Japan) | Cross sectional study | | Clinical examination Patient interview | HD patients with diabetic nephropathy (DN) and chronic glomerulonephritis (CGN) Control group subjects | Total 204 Control: n=106 (69M/37F) DN: n=29 (23M/6F) CGN: n=69 (43M/26F) | Control: 62.9 ± 2.5 DN: 60.5 ± 6.1 CGN: 61.4 ± 5.0 | DN: 9.5 ± 9.1 years CGN: 17.1 ± 11.0 years | N/A |
| Yazdi. FK (2013) | Middle East (Iran) | Clinical trial | | Cinical examination Patient interview Biochemical testing | HD Patients | HD patients: n=77 (52M) | 44.35 ± 17.2 years (range 14–88 years) | N/A | Gingivitis: no CAL  Mild Periodontitis  Moderate Periodontitis  Severe Periodontitis   Generalised Chronic Periodontitis: >30% periodontal involvement Localized Clinical Periodontitis: <30% of the sites involved |
| de Souza. CM (2014) | South America (Brazil) | Case control study | | Cinical examination Patient interview Biochemical testing | HD patients with periodontitis with and without treatment  and HD patients without periodontitis | Total 122 (79M/43F)  Untreated: 30 (19M/11F) Treated: 43 (28M/15F) No CP: 49 (32M/17F) | Total 50 ± 13  Untreated: 59 ± 11 Treated: 50 ± 11  No CP: 45 ± 12 | Total: 44 ± 41 months Untreated:38 ± 36  Treated:50 ± 46 months  No CP: 43 ± 41 months | CP: CAL ≥5 mm in ≥3 teeth in ≥2 quadrants |
| Hajian-Tilaki. A (2014) | Middle East (Iran) | Cross sectional study | | Clinical examination Patient interview | HD Patients | n=145 77 M (53.1%) and 68 F (63.9%) | 58.17 ± 17.76 years | 49.33 ± 46.99 months | N/A |
| Rodrigues. VP (2014) | South America (Brazil) | Cross sectional study | | Clinical examination Patient interview Biochemical testing | HD patients | n=96 individuals (45 M and 51 F) | 39.8 ± 13.2 years | 45.6 ± 33.1 months | Periodontitis: ≥2 sites with CAL ≥4 mm and/or ≥2 sites with PD ≥5 mm.  Prevalence of periodontitis was 59.4% |
| Zhou. D (2014) | China (Asia) | Case control study | | Clinical Examination | HD patients | Total n=306 HD patients n=102(59 M and 43 F) Control group: n=204 patients (118 M and 86 F) | HD: 58.4 ± 14.1 years Control: 59.3 ± 11.6 years | 1 to <2 years: 25 (24.5%) 2 to 5 years: 46 (45.1%) >5 years: 31 (30.4%) | CPI 0: no clinical signs CPI 1: gingivitis CPI 2: early periodontitis CPI 3: moderate periodontitis  CPI 4:severe periodontitis |
| Fang. F (2015) | Asia (China) | Randomised Control Trial | | Clinical Examination Biochemical testing | HD patients | Total n=97 Intervention: 48(20F) Control: 49 (22F) | Intervention: 53.71 ± 5.89 Control: 55.53 ± 6.74 | Intervention: 26.50 months Control: 29.00 months | Chronic Periodontitis: ≥ 1 mm mean CAL |
| Huang. ST (2015) | Asia (Taiwan) | Cohort study (Retrospective) | | Clinical record analysis | HD patients with periodontal treatment Control: HD patients without periodontal treatment | Total = 8902 Treatment = 4451 (2425F, 2026M)  Control =4451 (2435F, 2016M) | Treatment: 58.2±12.2  Control: 58.3±13.7 | 2.47 ± 2.24 years and 2.50 ± 2.25 years for the treatment and comparison cohorts | N/A |
| Limeres. J (2016) | Europe (Portugal) | Case control study | | Clinical Examination | HD patients | HD = 44 (26 F, 18 M) Healthy Control = 44 | 69.8 ± 10.0 years | N/A | N/A |
| Palmer. SC (2016) | Multinational  Europe (France, Hungary, Italy, Poland, Portugal and Spain)   South America (Argentina) | Cohort study | | Clinical examination Patient interview | HD patients | Total n=4205 (2426M/1779F)  Argentina n=1744 (41.5%) France n=48 (1.1%) Hungary n=550 (13.1%) Italy n=593 (14.0%) Poland n=319 (7.6%) Portugal n=762 (18.1%) Spain n=189 (4.4%) | 61.6±15.6 | 77.5±59.1 months | CPI 0: normal CPI 1: gingival bleeding CPI 2: calculus CPI 3: shallow periodontal pocket of 3.5–5.5 mm CPI 4: deep periodontal pocket of ≥5.5 mm |
| Schmalz. G (2016) | Europe (Germany) | Cross sectional study | | Clinical examination PCR Patient interview Biochemical testing | HD patients | n=35 patients.  40% (14) F | 56.4 ± 11.1 (29–79) | N/A | Page and Eke periodontitis classifications: No/mild periodontitis  Moderate Severe  Moderate (40%), severe (53%), no/mild (6.7%) |
| Schmalz. G (2016) | Europe (Germany) | Cross sectional study | | Multiple: Clinical Examination, patient interview | HD patients Kidney Transplant (KTx) patients who underwent KTx at least 5 years before Healthy Controls (HC) | HD group: 87 patients (37.9%F) KTx group 39 (51.3%F)  HC group: 91 (65.9%F) patients | HD group: 60.98 ± 14.01 years KTx group 56.51 ± 11.56 years HC group: 58.31 ± 9.91 years | HD therapy for at least 5 years | Page and Eke periodontitis classifications: No/mild periodontitis  Moderate Severe |
| Cotič. J (2017) | Europe (Slovenia) | Cross sectional study | | Multiple: Clinical examination, patient interview, biochemical testing | HD patients | 111 (68M) | 63 | 3.29 years | CPI 0: normal (only 1 patient) CPI 1: gingival bleeding (5.3% of patients) CPI 2: calculus (42.1%) CPI 3: shallow periodontal pocket of 3.5–5.5 mm (38.2%) CPI 4: deep periodontal pocket of ≥5.5 mm (13.2%) |
| Hou. Y (2017) | Asia (China) | Cohort study (retrospective ) | | Multiple: Clinical Examination, biochemical testing | HD patients | n = 136 | 50.8 ± 15.3 years | N/A | Periodontitis: gingival haemorrhage, formation of periodontal pocket, periodontal abscesses or periodontal abscess overflow, loose teeth, GR, or atrophy |
| Kim. YJ (2017) | South America (Brazil) | Cross sectional study | | Clinical Examination | HD patients | 115 total (71M, 44F) | 47.30±18.35 | 3.43±3.28 years | American Academy of Periodontology in mild, moderate and advanced periodontitis |
| Ruospo. M (2017) | Multinational: Europe South America | Cohort study | | Clinical Examination | HD patients | Total = 3338 Moderate/severe periodontitis= 1355 (852M) No/mild periodontitis = 1983 (1116M) | Moderate/Severe: 61.7 No/mild: 57.3 After: 61.8 | Moderate/Severe: 84.6 months No/mild: 75.1 months After: 82.5 months | None/mild periodontitis: CPI 0–2 Moderate to severe: CPI 3–4 |
| Schmalz. G (2017) | Europe (Germany) | Cross sectional study | | Multiple: Clinical Examination, biochemical testing | Patients undergoing HD | Total = 159 HD with no DM = 93 (59M) HD patients witb DM = 66 (43M) | No DM:66.7 ± 13.0  DM: 70.5 ± 10.2 | No DM:4.4 ± 4.1 Years  DM: 3.3 ± 2.7 years | American Academy of Periodontology/Centers for Disease Control and Prevention (AAP/CDC) case definitions of 2007: (severe periodontitis; (moderate periodontitis; or no/mild periodontitis |
| Camacho-Alonso. F (2018) | Europe (Spain) | Cross sectional study | | Multiple: clinical examination, patient interview | HD patients and healthy controls | n = 240 subjects, 157 M (65.42%) and 83 F (34.58%) HD patients = 120 (82 M,38 F)  Healthy controls = 120 (75 M, 45 F) | Overall mean age of 59.22 ± 17.69.  Stage 5 CRF patients mean age of 69.90 ± 11.61 years,  Healthy controls mean age of 67.71 ± 8.96 years | <1 year (n = 14)  1–2.9 years (n = 13)  3–4.9 years (n = 27)  5–9.9 years (n = 43)  >10 years (n = 23) | Number of remaining teeth and missing teeth, bleeding index, CPITN (CPITN), CAL (CAL), probe depth (PD), number of pockets ≥4 mm, number of pockets ≥6 mm, and the classification of periodontal disease as either healthy, mild, moderate, or severe. |
| Iwasaki. M (2018) | Asia (Japan) | Cohort study (Prospective) | | Cinical Examination | HD patients | Total: 211 (131 M, 80 F)  With Periodontal Disease: 92 (61 M, 31 F) Without Periodontal Disease: 119 (70 M, 49 F) | Total: 64.4±12.9  With periodontal disease: 69.5±10.6  Without periodontal disease: 60.4±13.2 | 86 months on average for whole population  With PD: 73.5 months Without: 102 months | Periodontal disease was defined as the presence of CAL of ≥4 mm in ≥30% of the probed sites |
| Kopić. V (2019) | Europe (Croatia) | Cross sectional study | | Multiple: Clinical examination, biochemical testing | control: patients with CKD stage III and IV, and  CKD stage V undergoing HD | 80 total control: 40 (23M/17F) HD: 40 (21M/19F) | control: 64.5 HD: 63 | Patient on HD for more than 3 months | N/A |
| Duan. X (2020) | Asia (China) | Cross sectional study | | Multiple: Clinical Examination, biochemical testing, PCR | patients undergoing HD three times per week | 208 total Healthy: 100 (50f 50M) HD: 108 (46F 62M) | HD: 46.19 ± 13.33 Healthy Controls: 46.78 ± 14.88 | N/A | Community Periodontal Index |
| Smojver. BK (2020) | Europe (Croatia) | Cross sectional study | | Clinical Examination | HD and PD patients | HD = 58 (31 M 27 F)  PD = 31 (16 M 15 F) | HD: 65 PD: 49 | HD 36 Months PD 12 Months | Periodontal inflamed surface area (PISA) calculated based on BOP, CAL and recession. |
| Mizutani. K (2020) | Asia (Japan) | Cohort study | | Multiple: Clinical Examination, biochemical testing | HD patients | HD = 207 (135 M) | 65.9 ± 12.1 | 64 (33, 115) months | The mean DI-S score was 0.99 ± 0.76. The number of patients with healthy, mild, moderate, or severe periodontal disease status was 13 (6.3%), 67 (32.4%), 82 (39.6%), and 45 (21.7%), respectively. |
| Oduncuoğlu. BF (2020) | Middle East (Turkey) | Cross sectional study | | Multiple: Clinical examination, patient interview | HD patients, patients with renal transplant and healthy controls | Total: 188 (104M/84F) Renal transplant 64 (44M/20F) HD 63 (39M/24F)  Control 61 (21M/40F) | Renal Transplant 37.90 ± 10.30 HD 40.98 ± 9.99  Control 37.10 ± 13.41 | HD therapy for at least 12 months | N/A |
| Pallos. D (2020) | South America (Brazil) | Cross sectional study | | Multiple: Clinical Examination, biochemical testing, PCR | Group 1 (clearance of creatinine > 75 mL/min): patients with no renal disease Group 2 (clearance of creatinine of 11-75 mL/min): patients with mild-moderate renal disease Group 3 (clearance of creatinine < 10 mL/min): patients with terminal renal disease—on HD | 131 total  Group 1 = 24 Group 2 = 67 Group 3 = 40 | N/A | N/A | Mild periodontitis: two or more interproximal sites with CAL ≥ 3 mm and two or more interproximal sites with PPD ≥ 4 mm (not on the same tooth) or one interproximal site with PPD ≥ 5 mm.  Moderate periodontitis: two or more interproximal sites with CAL ≥ 4 mm (not on the same tooth) or two or more interproximal sites with PPD ≥ 5 mm (not on the same tooth) Advanced periodontitis = two or more interproximal sites with CAL ≥ 6 mm (not on the same tooth) and one or more interproximal sites with PPD ≥ 5 mm |
| Kahar. P (2021) | North America (Florida) | Cross sectional study | | Multiple: Clinical Examination, patient interview | HD patients | HD = 93 (56 M 37 F) | 64.81 ± 12.9 | 4.34 ± 6.6 Years | Community periodontal index |
| Misaki. T (2021) | Asia (Japan) | Cohort study (prospective) | | Multiple: Clinical Examination, biochemical testing | Patients undergoing HD | HD = 80 (48 M 32 F) | 67.3 ± 12.2 | 7.6 ± 5.9 years | Periodontal statuses were assessed by measurement of PPD, in accordance with World Health Organization guidelines |
| Abou-Bakr. A (2022) | Africa (Egypt) | Cross sectional study | | Multiple: Clinical examination, biochemical testing | HD patients | HD = 263 (165 M and 98 F) | 48.12 | 16.01 months | N/A |
| Chung. WC (2022) | Asia (Taiwan) | Randomised Control Trial | | Multiple: Clinical Examination, biochemical testing, PCR | HD patients | Treatment: 7 (5M/2F) Control: 7 (5M/2F) | Total: 61±12 Treatment: 63±8 Control: 60±16 | Total: 46±59 months Treat: 60±81 Control: 31±20 | Centers for Disease Control and Prevention-American Academy of Periodontology (CDC-AAP) criteria. Periodontitis: presence of one or more teeth with pockets ≥4 mm. Moderate periodontitis: one or more sites with PD of 4 to 6 mm. Advanced periodontitis: one or more sites with PD >6 mm |
| Lu. H (2022) | Asia (China) | Case control study | | Multiple: Clinical Examination, biochemical testing | HD patients and healthy controls | HD = 63 Healthy Controls = 75 | N/A | nil, Treatment of HD for 3 times/week for 4h each session | Centers for Disease Control and Prevention-American Academy of Periodontology (CDC-AAP) criteria |
| Parkar. SM (2012) | Asia (India) | Cross sectional study | | Clinical Examination | HD patients and Healthy Controls | HD = 152 Healthy Control = 152 (75% M 25% F) | HD: 37.33±11.86 Control: 37.25±11.93 | The dialysis group was divided into four subgroups: (1) dialysis for less than 3 months, (2) dialysis for 4–6 months, (3) dialysis for 7–9 months, and (4) dialysis for 10–12 months | Community periodontal index CPI |
| Tavakoli. M (2016) | Middle East (Iran) | Cross sectional study | | Multiple: Clinical Examination, clinical record analysis | HD patients and Healthy Controls | n = 75 HD = 50 patients, further separated in those with metabolic syndomes (25) and those not (25) Healthy Controls = 25 | N/A | HD patient more than 5 years | Periodontal conditions determined by radiography, gingival index, PPD, and bleeding index |
| Tawfig. A (2016) | Middle East (Saudi Arabia) | Cross sectional study | | Multiple: Clinical Examination, biochemical testing | Healthy controls, patients on predialysis, on haemodialysis and post transplant | n = 120 (67M 53F) Each group had 30 participants | 41.90 ± 12.08 | N/A | Not defined but was classified into no, mild, moderate and severe periodontitis |
| Veisa. G (2016) | Europe (Romania) | Cohort study | | Multiple: Clinical Examination, biochemical testing | HD patients | HD = 200 (101 M) | 54.11±14.37 | 5.6±5.2 years | Periodontal Disease Index (PDI) |
| Iwasaki.M (2017) | Asia (Japan) | Cross sectional study | | Multiple: Clinical Examination, patient interview | HD pt catagorised into perio grades | Total 188 (118M/70F) | 63.6±12.8 | 7 years | Centers for Disease Control and Prevention/American Academy of Periodontology periodontitis case definitions to categorize the participants into the following three groups: severe, moderate, and no/mild periodontitis |
| Naghsh.N (2017) | Middle east (Iran) | Cross sectional study | | Multiple: Clinical Examination, biochemical testing | Patients undergoing HD | HD = 57 (37M/20F) | 47.9±14.6 | 48.7±47.5 months | Criteria by the American Academy of Periodontology: a minimum of two sites with CAL ≥ 4mm at the interproximal area or minimum of 2 interproximal areas with PD ≥ 5mm in different teeth |
| Sharma.L (2018) | Asia (India) | Retrospective cohort study | | Multiple: Clinical Examination, biochemical testing | Healthy controls and Patients on HD | n = 100 HD = 50 (47M) Control = 50 (5M) | HD:36.2±11.1 Control:35.9±6.64 | N/A | Severe: ≥2 interproximal sites with CAL ≥4 mm (not on the same tooth) And ≥1 interproximal sites with PD ≥5 mm Moderate: ≥2 interproximal sites with CAL ≥4 mm (not on the same tooth) OR ≥2 interproximal sites with PD ≥5 mm (not on the same tooth) Mild: Neither moderate or severe |
| Rapone.B (2019) | Europe (Italy) | Cohort study | | Multiple: Clinical Examination, biochemical testing | HD patients | 66 periodontitis patients undergoing HD treatment | 30-54 | 38.5 months | American Academy of Periodontology |
| Kovačević.P (2021) | Europe (Croatia) | Cross sectional study | | Multiple: Clinical Examination, biochemical testing | HD patients | 53 patients on chronic HD | 69 | ≥ 6 months | Interdental CAL is detectable at ≥2 non-adjacent teeth, or Buccal or oral CAL ≥3 mm with pocketing >3 mm is detectable at ≥2 teeth |
| Vrinda.S.A (2021) | Asia (India) | Clinical Trial - interventional study | | Multiple: Clinical Examination, biochemical testing | Chronic Periodontitis (CP) with various time duration under HD | 60 CP separated by HD duration: 20 HD< 1 year, 20 HD>1year and 20 no HD but are systematically healthy  Group 1 16M 4F  Group 2: 17M 3F  Group 3: 7M 13 F | Group 1: 51.15±9.66 Group 2: 49.90±1.20 Group 3: 41.35±8.79 | Group 1: chronic periodontitis undergoing HD < 1 year Group 2 : CP HD> 1 year Group 3: CP no HD | 30% of teeth were affected with attachment loss ≥ 5mm along with occurrence of BOP |
| Dembowska.E (2022) | South America (Brazil) | Cross sectional study | | Multiple: Clinical Examination, patient interview | HD patients and Control group | 100 HD patient 42F, 58M 100 control:43F 57M | HD: 55 years ±16.43 Control: mean age of 52 years (±15.46) | ≥ 3 months | Periodontitis classified according to Page and Eke: presence of one or more teeth with pockets ≥4 mm. Moderate periodontitis: one or more sites with PPD of 4 to 6 mm. Advanced periodontitis: one or more sites with PPD >6 mm |
| Dembowska.E (2022) | South America (Brazil) | Cross sectional study | | Clinical Examination | HD patients and healthy controls | 100 patients undergoing HD (42F, 58M) , 100 patients without CKD (43F, 57M), | HD: 55 ±16.43 Control: 52 years ±15.46 | ≥ 3 months | Periodontitis classified according to Page and Eke: presence of one or more teeth with pockets ≥4 mm. Moderate periodontitis: one or more sites with PPD of 4 to 6 mm. Advanced periodontitis: one or more sites with PPD >6 mm |
| Ghalwash.D.M (2022) | Africa (Egypt) | Cross sectional study | | Multiple: Clinical Examination, biochemical testing | Systemically healthy patients with periodontitis, ESRD patients undergoing HD who had gingivitis, HD ESRD patients with periodontitis | Group 1 = 15 patients (5 M and 10 F), Group 2 =15 (11 M and 4 F), Group 3: 15 (7 M and 8 F) | Group 1: 46.7±8.7  Group 2: 33.3±4.9  Group 3: 52.9 ± 8.1 | ≥ 3 months | Stage II periodontitis: 3–4 mm interdental CAL, coronal radiographic bone loss 15–33% that is mostly horizontal, no tooth loss because of periodontitis, and PPD ≤ 5 mm |
| Kotecha.K (2022) | Europe (England) | Cross sectional study | | Clinical Examination | HD patients | HD = 118 | Healthy gums/gingivitis = 54, patients with periodontitis = 63 | Range: 0 – 20 years | 2017 World Workshop on the Classification of Periodontal and Peri-Implant Diseases and Conditions. |
| Raeesi.V (2023) | Middle East (Iran) | Clinical Trial - interventional study | | Clinical Examination | HD patients and healthy controls | HD = 30 (15F, 15M) Healthy controls = 30 (17F, 13M) | HD: 43 ± 9.66 Healthy : 32.4 ± 10.28 | > 3 months | Newman and Carranza's Clinical Periodontology 2019 |
| Mikami.R (2023) | Asia (Japan) | Cross sectional study | | Multiple: Clinical Examination, biochemical testing | HD patients | HD = 254 (167 M) | 67.4±12.1 | 6.7±6.0 years | American Academy of Periodontology Classification: periodontal healthy, mild, moderate, severe |

Abbreviations: HD = Haemodialysis, PD = Peritoneal Dialysis, ESRD = End stage renal disease, CAPD: Continuous ambulatory peritoneal dialysis (CAPD), CRF = Chronic renal failure, DM = Diabetes Mellitus PPD = Pocket Probing Depth, LOA = Loss of attachment, CAL = Clinical Attachment Loss PI = Plaque Index, API = Approximal Plaque Index, SBI = Sulcus Bleeding Index, BOP = Bleeding on Probing, PDI = Periodontal Disease Index, PBI = Papillary Bleeding Index, CPI = Community Periodontal Index, CPITN = Community Periodontal Index of Treatment Needs, CRP = C-reactive protein, M = Males, F = Females, NSPT = Non-surgical periodontal treatment

Supplementary Table 1: Summary of included articles

| First Author (year of publication) | Region (Country) | Study Design | Periodontal Case Definitions | Measured outcomes | Data Collection Methods | Target population | Study size (gender) | Mean Age (years) | Mean dialysis time |
| --- | --- | --- | --- | --- | --- | --- | --- | --- | --- |
| Fang. F (2015) | Asia (China) | Randomised Control Trial | AAP 1999 [1] | Periodontal: PI, BOP, PPD, GR, CAL Biochemical: TNF-a, IL-6, CRP | Multiple: Clinical examination, Biochemical testing | HD patients with chronic periodontitis | Total: 97 Intervention: 48 (28M, 20F) Control: 49 (27M, 22F) | Intervention: 53.71 ± 5.89 Control: 55.53 ± 6.74 | Intervention: 26.5 months Control: 29 months |
| Yazdi. FK (2013) | Middle East (Iran) | Clinical trial | AAP 1999 [1] | Periodontal:number of teeth, PPD, GR, CAL, BI, PI Other: CRP | Multiple: Clinical examination, Biochemical testing | HD Patients | HD:77 (52M, 25F) | 44.35 ± 17.2 years | N/A |
| Iwasaki. M (2018) | Asia (Japan) | Cohort study (Prospective) | AAP 1999 [1] | Periodontal: CAL, OHI, | Cinical Examination | HD patients | Total: 211(131M, 80F) With PD: 92 (61M, 31F) Without PD: 119 (70M, 49F) | Total: 64.4±12.9 With PD: 69.5±10.6  Without PD: 60.4±13.2 | Total: 86 months  With PD: 73.5 months Without: 102 months |
| Hou. Y (2017) | Asia (China) | Cohort study (retrospective) | AAP 1999 [1] | Periodontal: PPD, gingnival haemorrhage, periodontal abscesses, loose teeth, gingival recession or atrophy | Multiple: Clinical examination, Biochemical testing | HD patients | HD: 136 (79M, 7F) | 50.8 ± 15.3 years | N/A |
| de Souza. CM (2014) | South America (Brazil) | Case control study | AAP 1999 [1] | Periodontal: GI, PI, CI, PDD, CAL Other: DMFt, oral hygiene | Multiple: Clinical examination, Biochemical testing | HD patients with periodontitis with and without treatment  and HD patients without periodontitis | HD: 122 (79M, 43F) | 50 ± 13 | 44 ± 41 months |
| Mikami.R (2023) | Asia (Japan) | Cross-sectional study | AAP 1999 [1] | Periodontal: PPD, CAL, BOP Other: Malnutrition, inflammation and atherosclerosisi syndrome | Multiple: Clinical examination, Biochemical testing | HD patients | HD: 254 (167M, 87F) | 67.4±12.1 | 6.7±6.0 years |
| Kim. YJ (2017) | South America (Brazil) | Cross-sectional study | AAP 1999 [1] | Periodontal: PI, GI, PPD, CAL | Clinical Examination | HD patients | n: 115 (71M, 44F) | 47.30±18.35 | 3.43±3.28 years |
| Naghsh.N (2017) | Middle east (Iran) | Cross-sectional study | AAP 1999 [1] | Periodontal: PPD, PI, BOP, GI, CAL | Multiple: Clinical examination, Biochemical testing | Patients undergoing HD | HD: 57 (37M, 20F) | 47.9±14.6 | 48.7±47.5 months |
| Brito. F (2012) | South America (Brazil) | Cross-sectional study | AAP 1999 [1] | Periodontal: PI, BOP, PPD, CAL | Multiple: Clinical examination Patient interview | CAPD Patients HD Patients Predialysis Patients Healthy Controls | Total:198 CAPD patients: 40 (10M, 30F) HD patients: 40 (21M, 19F ) Predialysis patients: 51 (29M, 22F) Controls: 67 (23M, 44F) | CAPD patients 52±12,  HD patients 50±10,  Predialysis patients 54±11  Controls 50±7 | 73.4 ± 56.2 months |
| de Souza. CM (2007) | South America (Brazil) | Cross-sectional study | AAP 1999 [1] | Peridontal: PPD, CAL, GI, PI, CI, mobility Other: CRP, vitamin D receptor gene polymorphism | Multiple: Clinical examination, Biochemical testing | Group 1: CKD without periodontitis, Group 2: no CKD with periodontitis, Group 3: ESRD on HD without periodontitis, Group 4: ESRD on HD with periodontitis | Total: 222 (Healthy: 59, without CKD with PD: 50, HD without PD: 50, HD with PD: 63 | 44.9 (range 23–77) | Without PD: 47.8,  With PD: 47.2 |
|  |  |  |  |  |  |  |  |  |  |
|  |  |  |  |  |  |  |  |  |  |
| Rapone.B (2019) | Europe (Italy) | Cohort study (prospective) | AAP 2017 [2] | Periodontal: PI, GI, PDD, CAL Other: serum albumin, creatinine, CRP | Multiple: Clinical examination, Biochemical testing | HD patients | HD: 66 | 30-54 | 38.5 months |
| Abou-Bakr. A (2022) | Africa (Egypt) | Cross-sectional study | AAP 2017 [2] | Other: Urea, creatinine Periodontal: PI, GI, PPD, CAL | Multiple: Clinical examination, Biochemical testing | HD patients | HD: 263 (165M, 98F) | 48.12 ±n8.8 | 16.01 ± 6.41 months |
| Ghalwash.D.M (2022) | Africa (Egypt) | Cross-sectional study | AAP 2017 [2] | Periodontal: Plaque, BOP, PPD, CAL Other: Fibroblast growth factor 23 | Multiple: Clinical examination, Biochemical testing | Systemically healthy patients with periodontitis, ESRD patients undergoing HD who had gingivitis, HD ESRD patients with periodontitis | Total: 45 (23M, 22F) Healthy with PD: 15 (5M, 10F), HD with gingivitis: 15 (11M, 4F), HD with PD: 15 (7M, 8F) | Healthy with PD: 46.7±8.7 HD with gingivitis: 33.3±4.9 HD with PD: 52.9 ± 8.1 | ≥ 3 months |
| Kotecha.K (2022) | Europe (England) | Cross-sectional study | AAP 2017 [2] (An adaptation) | Other: Haemoglobin A1C, Albumin, CRP, parathyroid hormone, oral hygiene | Multiple: Clinical Examination, Biochemical testing | HD patients | HD: 118 | No PD: 54 With PD: 63 | 0-20 years |
| Kovačević.P (2021) | Europe (Croatia) | Cross-sectional study | AAP 2017 [2] | Periodontal: API, PPD, PBI, BOP, CAL, GR Other: body composition, CRP, albumin | Multiple: Clinical examination, Biochemical testing | HD patients | HD: 53 | 69 | ≥ 6 months |
|  |  |  |  |  |  |  |  |  |  |
| Castillo. A (2007) | Europe (Spain) | Cross-sectional study | Arbes et al 1999 [3] | Periodontal: LPA, GBI, PI Other: calcium, alkaline phosphatase, glucose, albumin, parathyroid hormone, periodontal pathogens | Multiple: Clinical examination, Biochemical testing | HD Patients Healthy Controls | HD: 52 (24M, 28F) Controls: 52 (24M, 28F) | HD: 61.5 Control: 59.8 | 43.4±38.96 months |
| Mizutani. K (2020) | Asia (Japan) | Cohort study (Prospective) | Page and Eke [4] | Periodontal: tooth mobility, BOP, PPD, CAL Other: P.gingivalis , hsCRP, high sensitivity C reactive protein, simplified debris index , frequency of toothbrushing, DMFT | Multiple: Clinical examination, Biochemical testing | HD patients | HD: 207 (135M, 72F) | 65.9 ± 12.1 | 64 months |
| Sharma.L (2018) | Asia (India) | Cohort Study (Restrospective) | Page and Eke [4] | Periodontal: CPI, BOP, PPD, CAL | Multiple: Clinical examination, Biochemical testing | Healthy controls, Patients on HD | HD: 50 (47M, 3F) Controls: 50 (5M, 45F) | HD:36.2±11.1 Control:35.9±6.64 | N/A |
| Lu. H (2022) | Asia (China) | Case control study | Page and Eke [4] | Periodontal: PI, GI, CI, PPD, CAL | Multiple: Clinical Examination, Biochemical testing | HD patients and healthy controls | HD: 63 (37M, 26F) Controls: 75 (40M, 35F) | 18-75 | N/A |
| Dembowska.E (2022) | South America (Brazil) | Cross-sectional study | Page and Eke [4] | Periodontal: PPD, CAL, GI, BOP | Clinical Examination | HD patients and healthy controls | HD: 100 (42F, 58M) Control: 100 (43F, 57M) | HD: 55 ±16.43 Control: 52 years ±15.46 | ≥ 3 months |
| Dembowska.E (2022) | South America (Brazil) | Cross-sectional study | Page and Eke [4] | Periodontal: PI, GI, PPD, CAL Other: WHOQOL-BREF | Multiple: Clinical Examination, Patient interview | HD patients and Control group | HD: 100 (42F, 58M) Control: 100 (43F, 57M) | HD: 55 ±16.43 Control: 52 years ±15.46 | ≥ 3 months |
| Pallos. D (2020) | South America (Brazil) | Cross-sectional study | Page and Eke [5] | Periodontal: PPD, CAL, GI, PI Other: Human herpesvirus | Multiple: Clinical examination, Biochemical testing | Group 1: clearance of creatinine > 75 mL/min, patients with no renal disease Group 2: clearance of creatinine of 11-75 mL/min, patients with mild-moderate renal disease Group 3: clearance of creatinine < 10 mL/min, patients with terminal renal disease on HD | Total: 131 No renal disease: 24 Mild-moderate renal disease: 67 HD: 40 | N/A | N/A |
| Iwasaki.M (2017) | Asia (Japan) | Cross-sectional study | Page and Eke [4] | Periodontal: CAL, PPD Other: medical outcomes study 36-item Short Form health Survey (SF-36), albumin, cholesterol, BMI, comorbidities | Multiple: Clinical Examination, Patient interview | HD patients | HD: 188 (118M, 70F) | 63.6±12.8 | 7 years |
| Schmalz. G (2017) | Europe (Germany) | Cross-sectional study | Page and Eke [4] | Peridontal: PPD, BOP, CAL Other: DMFT, periodontal pathogens, saliva test | Multiple: Clinical examination, Biochemical testing | Patients undergoing HD | Total: 159 HD with no DM: 93 (59M, 34F) HD patients with DM: 66 (43M, 23F) | No DM:66.7 ± 13.0  DM: 70.5 ± 10.2 | No DM:4.4 ± 4.1 Years  DM: 3.3 ± 2.7 years |
| Schmalz. G (2016) | Europe (Germany) | Cross-sectional study | Page and Eke [4] | Periodontal: PBI, PPD, BOP, CAL Other: DMFT, periodontal pathogens | Multiple: Clinical examination, Biochemical testing, Interview | HD patients, Kidney transplant (KT) patients | Total: 70 HD: 35 KT: 35 | HD: 56.4 ± 11.1 (29–79) KT: 55.4 ± 10.9 | HD: 5.5 ± 6.4 years |
| Schmalz. G (2016) | Europe (Germany) | Cross-sectional study | Page and Eke [4] | Periodontal: PPD, CAL Other: DMFT, OHIP G14 | Multiple: Clinical Examination, Interview | HD patients Kidney Transplant (KT)  Healthy Controls (HC) | HD: 87 (37.9%F) KT: 39 (51.3%F)  Control: 91 (65.9%F) | HD: 60.98 ± 14.01  KT: 56.51 ± 11.56  Control: 58.31 ± 9.91 | HD for at least 5 years |
| Rodrigues. VP (2014) | South America (Brazil) | Cross-sectional study | Page and Eke [4] | Periodontal: PPD, CAL, GBI, GI, PI Other: albumin, transferrin, ferritin, phosphorus, potassium, haemoglobin | Multiple: Clinical examination, Biochemical testing, Patient interview | HD patients | HD: 96 (45M and 51F) | 39.8 ± 13.2 years | 45.6 ± 33.1 months |
| Smojver. BK (2020) | Europe (Croatia) | Cross-sectional study | Periodontal inflamed surface area (PISA) [6] | Periodontal: BOP, CAL, GR | Clinical Examination | HD and PD patients | HD: 58 (31M, 27F)  PD: 31 (16M, 15F) | HD: 65 PD: 49 | HD: 36 Months PD: 12 Months |
| Veisa.G (2017) | Europe (Romania) | Cross-sectional study | PDI [7] | Periodontal: GP, PI, CI Other: QOL (SF-36) | Multiple: Clinical examination, Patient interview | HD patients | HD: 101 (44M, 57F) | 52.5 ±14.3 | 6.7±5.6 years |
| Veisa. G (2016) | Europe (Romania) | Cohort study (prospective) | PDI [7] | Periodontal: PDI Other: CRP, IL-6, TNFa, nutritional markers (BMI, serum albumin) | Multiple: Clinical examination, Biochemical testing | HD patients | HD: 200 (101M, 99F) | 54.11±14.37 | 5.6±5.2 years |
| Chen L.P (2011) | Asia (Taiwan) | Cross-sectional study | PDI [7] | Periodontal: PI, GI, PDI | Clinical examination | HD Patients | HD: 253 (117M, 136DF) | 58.8 ± 0.8 | 48.8 ± 3.2 months |
| Chen. LP (2011) | Asia (Taiwan) | Cross-sectional study | PDI [7] | Periodontal: PI, GI, PDI Other: causes of death | Multiple: Clinical examination, Clinical record analysis | HD Patients | HD: 253 (117M, 136F) | 58.8 ± 0.8 | 48.8 ± 3.2 months |
| Chen. LP (2006) | Asia (Taiwan) | Cross-sectional study | PDI [7] | Periodontal: PI, GI, PPD, CAL Other: Serum albumin, CRP, blood urea nitrogen, lymphocytes, transferrin, protein catabolism rate, total cholesterol | Multiple: Clinical examination, Biochemical testing | HD Patients | HD: 253 (117M, 136F) | 58.8 ± 0.8 | 48.6 ± 3.2 months |
| Kadiroglu. AK (2006) | Middle East (Turkey) | Cross-sectional study | PDI [7] | Periodontal: GI, PPD, PPD, CAL Other: leukocytes, albumin, cholesterol, parathyroid hormone, inflammatory markers (ferritin, ESR, CRP) | Multiple: Clinical examination, Biochemical testing | HD Patients | Total: 41 High CRP: 21 (9M, 12F) Normal CRP: 20 (10M, 10F) | High CRP: 43.1±13.1 Low CRP: 39.2±14.6 | HIgh CRP: 23.4±23.4 months  Normal CRP: 26.4±25.2 months. |
| Raeesi.V (2023) | Middle East (Iran) | Clinical trial | Newman and Carranza's Clinical Periodontology 2019 [9] | Periodontal: PI, PPD, CAL | Clinical Examination | HD patients and healthy controls | HD: 30 (15M, 15F) Controls: 30 (13M, 17F) | HD: 43 ± 9.66 Controls : 32.4 ± 10.28 | > 3 months |
| Kshirsagar. AV (2007) | North America (United States) | Cross-sectional study | ≥60% of sites with CAL ≥4 mm | Periodontal : PPD, gingival recession, GI, BOP, CAL Other: albrumin, CRP | Multiple: Clinical examination, Biochemical testing | HD patients | HD: 154 (85M, 69F) | 54.6±13.3 | 4.0±3.2 years |
| Takeuchi. Y (2007) | Asia (Japan) | Case control study | Four sites with PPD >4mm | Periodontal: PI, GI, PPD Other: saliva test, cariogenic bacteria and periodontal bacteria | Multiple: Clinical examination, Biochemical testing | Patients with renal disease (non-haemodialysis, haemodialysis) and healthy controls | Total: 143 Renal disease: 40 HD: 41 Control: 62 | Renal disease and HD patients: 61.6 ± 9.84 Control: 57.6 ± 15.8 | 10.24 ± 8.10 years |
| Dağ. A (2010) | Middle East (Turkey) | Cross-sectional study | PPD ≥ 3mm | Periodontal: PI, GI, PPD Other: CRP, leukocytes, albumin, TNF-a | Multiple: Clinical examination, Biochemical testing | HD patients, Healthy controls | Total: 86 HD: 43 (20M, 23F) Control: 43 (21M, 22F) | HD: 42.02±17.05 Control: 41.11 ± 16.13 | 36.0±1.70 months |
| Misaki. T (2021) | Asia (Japan) | Cohort study (Prospective) | PPD ≥4 and≥6 mm | Periodontal: PPD Other: DMFT, aortic calcification Index | Multiple: Clinical examination, Biochemical testing | HD patients | HD: 80 (48M, 32F) | 67.3 ± 12.2 | 7.6 ± 5.9 years |
| Baioni. CS (2008) | South America (Brazil) | Case control study | CAL≥5 mm, in at least three teeth, in at least two quadrants | Periodontal: PPD, CAL, GI, PI, Calculus, mobility Other: polymorphism (OPG gene) | Multiple: Clinical examination, Biochemical testing | Group 1: healthy patients; Group 2, without CKD and with periodontal disease; Group 3, with CKD and without periodontal disease; Group 4, with CKD and periodontal disease | Total: 224 Group 1: 60 (43F,17M) Group 2: 50 (33F/17M) Group 3: 50 (16F/34M) Group 4: 64 (23F/41M) | Group 1: 37.8±9.6 Group 2: 40.8±9.4  Group 3: 45.2±12.9 Group 4: 54.5±12.2 | Group 3: 47.8 ± 48.0 months  Group 4: 47.2 ± 43.4 months |
| Kshirsagar. AV (2009) | North America (United States) | Cohort study (Retrospective) | Moderate-to-severe periodontitis: 2 or more teeth with at least 6 mm interproximal attachment level and at least 1 site with probing depth >5 mm. Mild or no periodontitis: absence of findings | Periodontal: attachment loss and PPD Other: CVD mortality, cardiac mortality, all cause mortality | Multiple: Clinical examination, Biochemical testing | HD patients | HD: 168 No/Mild Perio: 100 (54M, 46F) Moderate/Severe: 64 (23M, 41F) | no/mild: 52.2 ± 13.0 Mod/Sev: 55.7 ±13.3 | No/Mild: 3.8 ± 2.7 years Mod/Sev: 4.0 ± 3.8 years |
| Tawfig. A (2016) | Middle East (Saudi Arabia) | Cross-sectional study | N/A. Classified into no, mild, moderate, severe peridontitis | Periodontal: GI, CAL, PSR Other: saliva | Multiple: Clinical examination, Biochemical testing | Healthy controls, patients on predialysis, HD and post transplant | HD: 120 (67M, 53F) | 41.90 ± 12.08 | N/A |
|  |  |  |  |  |  |  |  |  |  |
| Vrinda.S.A (2021) | Asia (India) | Clinical trial | 30% of teeth were affected with attachment loss ≥ 5mm along with occurrence of BOP | Periodontal: GI, PI, PPD, BOP, CAL Other: CRP, iron, hemoglobin, erythrrocyte sedimentation rate (ESR) | Multiple: Clinical Examination, Biochemical testing | Chronic Periodontitis (CP) with various time duration under HD | HD: 60 (40M, 20F) | HD < 1 year: 51.15±9.66 HD > 1 year: 49.90±1.20 no HD: 41.35±8.79 | N/A |
| Chung. WC (2022) | Asia (Taiwan) | Randomised Control Trial | N/A | Periodontal: PI, PPD, GR, CAL, BOP Biochemical: CRP, GCF, TNF-α, IL-1β, IL-6, Pentraxin 3 (PTX3) Microbiological | Multiple: Clinical examination, Biochemical testing | HD patients | Total: 14 Treatment: 7 (5M, 2F) Control: 7 (5M, 2F) | Total: 61±12 Treatment: 63±8 Control: 60±16 | Total: 46±59 months Treat: 60±81 Control: 31±20 |
| Huang. ST (2015) | Asia (Taiwan) | Cohort study (Retrospective) | N/A | Periodontal: periodontal treatment Other: hospitalisation | Clinical record analysis | HD patients with periodontal treatment Control: HD patients without periodontal treatment | Total: 8902 Treatment: 4451 (2026M, 2425F)  Control: 4451 (2016M, 2435F) | Treatment: 58.2±12.2  Control: 58.3±13.7 | Treatment: 2.47 ± 2.24 years Control: 2.50 ± 2.25 years |
|  |  |  |  |  |  |  |  |  |  |
| Parente.I.T (2018) | South America (Brazil) | Case control study | N/A | Periodontal: CPI, PI, BOP, CAL | Multiple: Clinical examination, Patient interview | HD patients | HD: 45 (68.8% M) Controls: 26 (42.3% M) | HD: 48.59 ± 17.73 Healthy: 37.88 ± 14.21 | N/A |
| Limeres. J (2016) | Europe (Portugal) | Case control study | N/A | Periodontal: supragingival plaque, calculus deposits, gingival inflammation, PPD, CAL Other: DMFT, saliva (creatinine, urea) | Multiple: Clinical examination, Biochemical testing | HD patients | HD: 44 (18M, 26F) Controls: 44 (18M, 26F) | HD: 69.8 ± 10.0 years Control: 69.3 ± 10.0 years | N/A |
| Zhou. D (2014) | China (Asia) | Case control study | N/A | Periodontal: CPI, CAL | Clinical Examination | HD patients | Total: 306 HD: 102 (59M, 43F) Control: 204 (118M, 86F) | HD: 58.4 ± 14.1 Control: 59.3 ± 11.6 | 1 to <2 years: 25 (24.5%) 2 to 5 years: 46 (45.1%) >5 years: 31 (30.4%) |
| Amara Swapna. L (2013) | Asia (India) | Case control study | N/A | Periodontal: Calculus, PPD, BOP Other: oral manifestations (dry mouth, odour, ulceration), DMFT | Clinical Examination | Diabetic and non diabetic patients on HD | Total: 97 (Nondiabetic: 50, Diabetic: 47) | non-diabetic: 55.34±10.56; diabetic: 53.78±11.77 | non-diabetic: 3.98±1.79 years  diabetic: 3.13±.1.41 years |
| Cengiz. MI (2009) | Middle East (Turkey) | Case Control Study | N/A | Periodontal: PI, GI, PPD, LPA Other: DMFT | Clinical Examination | HD Patients Healthy Controls | Total: 109 HD: 68 (37M, 31F) Control: 41 (21M, 20F) | HD:47.85 ± 14.61  Control: 44.80 ± 10.22 | N/A |
| Frankenthal. SF (2002) | Middle East (Israel) | Case control study | N/A | Periodontal: PI, FI, PPD, CAL. Other: PTH | Clinical Examination | HD patients with Secondary Hyperparathyroidism (HPT) and healthy controls | Total: 70 HD: 35, control: 35) | 33 | N/A |
| Gavaldá.C (1999) | Europe (Spain, UK) | Case control study | N/A | Periodontal: PPD, gingival recession. Other: oral mucosa, saliva flow | Clinical Examination | HD patients vs healthy controls | Total: 158 HD: 105 (53M, 52F) Controls: 53 (29M, 24F) | HD: 58.9±14.9 years Controls: 55.76±10.7 | 59.8±43.9 months. |
| Noor, H (2023) | Asia (Pakistan) | Cross-sectional study | N/A | Periodontal: BOP, calculus, PPD 4-5mm Other: DMFT | Clinical examination | HD patients (CKD 3,4,5)  Exclusion: Dialysis for reasons other than CRF, systemic illness | n: 32 (55.6% M 44.6%F) | 33.08 ± 17.042 | N/A |
| Kahar. P (2021) | North America (Florida) | Cross-sectional study | N/A | Periodontal: CPI Other: GOHAI-12, DMFT, occluding pairs of teeth | Multiple: Clinical Examination, Patient interview | HD patients | HD: 93 (56M, 37F) | 64.81 ± 12.9 | 4.34 ± 6.6 Years |
| Duan. X (2020) | Asia (China) | Cross-sectional study | N/A | Periodontal: CPI Other: DMFT, salivary microbiome | Multiple: Clinical examination, Biochemical testing | patients undergoing HD three times per week | Total: 208  HD: 108 (62M, 46F) Control: 100 (50M, 50F) | HD: 46.19 ± 13.33 Controls: 46.78 ± 14.88 | N/A |
| Oduncuoğlu. BF (2020) | Middle East (Turkey) | Cross-sectional study | N/A | Periodontal: PI, GI, PPD Other: DMFT, Short Form-36 (SF-36), OHIP-14, OHRQoL-UK | Multiple: Clinical examination, Patient interview | HD patients, patients with renal transplant and healthy controls | n: 188 (104M, 84F) Renal transplant: 64 (44M, 20F) HD: 63 (39M, 24F)  Control: 61 (21M, 40F) | Renal Transplant: 37.90 ± 10.30 HD: 40.98 ± 9.99  Control: 37.10 ± 13.41 | HD therapy for at least 12 months |
| Kopić. V (2019) | Europe (Croatia) | Cross-sectional study | N/A | Periodontal: PPD, GR, API, PBI Other: CRP, IL-6, IL-17A, TNF-a | Multiple: Clinical examination, Biochemical testing | Control: patients with CKD stage III and IV, and  CKD stage V undergoing HD | Total: 80 HD: 40 (21M, 19F) Control: 40 (23M/17F) | HD: 63 Control: 64.5 | > 3 months |
| Camacho-Alonso. F (2018) | Europe (Spain) | Cross-sectional study | N/A | Periodontal: BI, CPITN, CAL, PPD, number of missing teeth Other: saliva test, OHIP-14, HADS | Multiple: Clinical examination, Patient interview | HD patients and healthy controls | Total: 240 (157M, 83F) HD patients: 120 (82M,38F)  Controls: 120 (75M, 45F) | 59.22 ± 17.69. | <1 year to >10 years |
| Cotič. J (2017) | Europe (Slovenia) | Cross-sectional study | N/A | Periodontal: API, SBI, CPITN Other: DMFT, CRP, periodontal pathogens | Multiple: Clinical examination, Interview, Biochemical testing | HD patients | HD: 111 (68 M, 43 F) | 63 | 3.29 years |
| Ruospo. M (2017) | Multinational: Europe South America | Cross-sectional study | N/A | Periodontal: PPD, CAL, BOP Other: all-cause mortality | Clinical Examination | HD patients | HD: 3338 | Moderate/Severe: 61.7 No/mild: 57.3 | Moderate/Severe: 84.6 months No/mild: 75.1 months |
|  |  |  |  |  |  |  |  |  |  |
|  |  |  |  |  |  |  |  |  |  |
| Ma.L (2016) | Asia (Bejing, China) | Cross-sectional study | N/A | Periodontal: PI, GI, CSI, PPD | Multiple: Clinical examination, Patient interview | HD patients and PD patients, healthy controls | Total: 127 HD: 49 (62.5%M) PD: 38 (60.5%M) Control: 40 (46.2%M) | HD group: 54.45±15.34 PD group: 59.29±15.00 Control: 54.38±11.96; | N/A |
| Palmer. SC (2016) | Multinational: Europe (France, Hungary, Italy, Poland, Portugal and Spain), South America (Argentina) | Cross-sectional study | N/A | Periodontal: PPD, CAL, BOP Other: DMFT, oral pain, dysgeusia, oral mucosal lesions, salivary flow rate, oral care | Multiple: Clinical examination, Questionnaire | HD patients | HD: 4205 (2426M, 1779F) | 61.6±15.6 | 77.5±59.1 months |
| Tavakoli. M (2016) | Middle East (Iran) | Cross-sectional study | N/A | Periodontal: GI, BI, PPD Other: metabolic syndrome | Multiple: Clinical Examination | HD patients and Healthy Controls | Total: 75 HD: 50 patients Controls: 25 | N/A | HD patient more than 5 years |
| Teratani. G (2013) | Asia (Japan) | Cross-sectional study | N/A | Periodontal: PPD, BOP, CAL Other: salivary flow, | Multiple: Clinical examination, Biochemical testing | HD patients with diabetic nephropathy (DN) and chronic glomerulonephritis (CGN) Control group subjects | Total: 204 Control: 106 (69M/37F) Diabetic nephropathy (DN): 29 (23M, 6F) Chronic glomerulonephritis (CGN): 69 (43M, 26F) | Control: 62.9 ± 2.5 DN: 60.5 ± 6.1 CGN: 61.4 ± 5.0 | DN: 9.5 ± 9.1 years CGN: 17.1 ± 11.0 years |
| Parkar. SM (2012) | Asia (India) | Cross-sectional study | N/A | Periodontal: CPI, LOA Other: simplified oral hygiene index | Clinical Examination | HD patients and Healthy Controls | HD: 152 (114M, 32F) Control: 152 (114M, 32F) | HD: 37.33±11.86 Control: 37.25±11.93 | N/A |
| Sekiguchi. RT (2012) | South America (Brazil) | Cross-sectional study | N/A | Periodontal: distance between cemento-enamel junction and gingival margin, PPD, CAL, BOP, GI, PI Other: DMFT | Clinical Examination | Group L: HD ≤ 36 months Group M: HD > 37 months | HD: 94 (51M, 43F) | 20-39 yrs: n=44 40-79 yrs: n=50 | 1-5 years |
| Siribamrungwong. M (2012) | Asia (Thailand) | Cross-sectional study | N/A | Periodontal: PI, PDI Other: Haemoglobin, urea nitrogen, leukocyte, creatinine, calcium, phosphate, albumin, cholesterol, parathyroid hormone, ferritin, CRP | Multiple: Clinical examination, Biochemical testing | HD Patients | HD: 30 (18M, 12F) | 62 ± 15 | N/A |
| Torres. SA (2010) | South America (Brazil) | Cross-sectional study | N/A | Periodontal: PI, GI, CI, GR, PPD Other: periodontal bacteria | Multiple: Clinical examination, Biochemical testing | HD patients with CRF and Healthy Controls with periodontitis | Total: 30 CRF: 16 (12M, 4F) Control: 14 (10M, 4F) | CRF: 41.7±7.2  Control: 41.4±7.6 | 29.1±22.4 months |
| Guzeldemir. E (2009) | Middle East (Turkey) | Cross-sectional study | N/A | Periodontal: PI, GI, PDD, BOP Other: OHRQOL (OHIP-14 and GOHAI), blood chemistry | Multiple: Clinical examination, Patient interview, Biochemical testing | HD patients | HD: 47 (23F, 24M) | mean age, 46.38 ± 15.10 years | 71.04 ± 1.16 months |
|  |  |  |  |  |  |  |  |  |  |
| Thorman. R (2009) | Europe (Sweden) | Cross-sectional study | N/A | Periodontal: BOP, LPA, PAI Other: oral mucosal lesions, DMFT | Clinical Examination | Predialysis, CAPD and HD | Total: 93 (62M, 31F) Predialysis: 59 CAPD: 19 HD: 15 | 61 (30-89) | >6 months |
| Borawski. J (2007) | Europe (Poland) | Cross-sectional study | N/A | Periodontal: GI, PBI, PI, CAL, CPI Other: CRP, serum albumin, leukocytes, cardiovascular disease, diabetes | Multiple: Clinical examination, Biochemical testing | HD Patients, CAPD Patients, Pre-Dialysis Patients Healthy Controls with advanced periodontitis, Healthy Controls | Total: 162 HD: 35 (69 M% 31% F) CAPD: 33 (58% M, 42% F) Pre-Dialysis CKD: 38 (47% M, 53% F) Healthy Controls with advanced PD: 26 (62% M, 38% F) Healthy Controls: 30 (50% M, 50% F) | HD: 56±11 CAPD: 51±11 Pre-Dialysis: 51±15 Healthy Controls with advanced periodontitis 49±7 Healthy Controls: 47±10 | HD: 14months (2-206) CAPD: 14months (3-72) |
| Cunha. FL (2007) | South America (Brazil) | Cross-sectional study | N/A | Periodontal: CAL, BOP, subgingival calculus Other: DMFT, TMJ | Multiple: Clinical examination, Clinical record analysis | CRF patients undergoing HD | HD: 160 (91M, 69F) | 59±12 (range 40-85) | 24 (11months-11years) |
| Duran. I (2004) | Middle East (Turkey) | Cross-sectional study | N/A | Periodontal: CPITN | Clinical Examination | HD patients and CAP\D patients | Total: 342 (299 HD 43 CAPD, 179 M, 163 F) | 43.7±15.5 | N/A |
| Marakoglu.I (2003) | Middle East (Turkey) | Cross-sectional study | N/A | Periodontal: GI, PI, PPD | Clinical Examination | HD patients and healthy controls | HD:36 (20M, 16F) Controls:36 (20M, 16F) | HD: 50.4±14.2 Control: 50.2±12.4 | N/A |
| Rahmati. MA (2002) | North America | Cross-sectional study | N/A | Periodontal: Periodontal pathogens Other: CRP, IgG antibody | Multiple: Clinical examination, Biochemical testing | HD patients | HD: 86 (46M, 40F) | 53.3 ± 16.1 | 22 months (range, 0 to 148 months) |
| Rahman. M.M (1992) | Middle East (Turkey) | Cross-sectional study | N/A | Peridontal: PPD, PI, BI, GI, PI | Clinical Examination | HD patients and patients with renal transplant and control patients | HD: 52 (41M, 11F) Control: 52 (41M, 11F) | HD: 32.25 Control: 32.25 | N/A |

Abbreviations: AAP: American Academy of Periodontology, API: Approximal Plaque Index, BOP: Bleeding on Probing, CAL: Clinical Attachment Loss, CAPD: Continuous ambulatory peritoneal dialysis (CAPD), CPI: Community Periodontal Index, CPITN: Community Periodontal Index of Treatment Needs, CRF: Chronic renal failure, CRP: C-reactive protein, CSI: Calculus surface index, DM: Diabetes Mellitus, F: Females, ESR: Erythrocyte sedimentation rate, ESRD: End stage renal disease, GBI: Gingival bleeding index, GP: Gingival and periodontal index, GR: Gingival recession, HADS: Hospital anxiety and depression scale, HD: Haemodialysis, IL-6: Interleukin-6, LOA: Loss of attachment, LPA: Loss of periodontal attachment, M: Males, NSPT: Non-surgical periodontal treatment, OHIP-14: Oral health impact profile, OHRQoL: Oral health-related quality of life, PAI: Periapical index, PBI: Papillary Bleeding Index PD: Periodontal Disease, PDI: Periodontal Disease Index, PI: Plaque Index, PPD: Pocket Probing Depth, SBI: Sulcus Bleeding Index, TNF-a (Tumour necrosis factor alpha,

**References**

1. Armitage, G.C., *Development of a Classification System for Periodontal Diseases and Conditions.* Annals of Periodontology, 1999. **4**(1): p. 1-6.

2. Caton, J.G., et al., *A new classification scheme for periodontal and peri-implant diseases and conditions – Introduction and key changes from the 1999 classification.* Journal of Periodontology, 2018. **89**(S1): p. S1-S8.

3. Arbes, S.J., Jr., G.D. Slade, and J.D. Beck, *Association between extent of periodontal attachment loss and self-reported history of heart attack: an analysis of NHANES III data.* J Dent Res, 1999. **78**(12): p. 1777-82.

4. Page, R.C. and P.I. Eke, *Case Definitions for Use in Population-Based Surveillance of Periodontitis.* Journal of Periodontology, 2007. **78**(7S): p. 1387-1399.

5. Eke, P.I., et al., *Update of the case definitions for population-based surveillance of periodontitis.* J Periodontol, 2012. **83**(12): p. 1449-54.

6. Park, S.Y., et al., *Periodontal inflamed surface area as a novel numerical variable describing periodontal conditions.* J Periodontal Implant Sci, 2017. **47**(5): p. 328-338.

7. Frankenthal, S., et al., *The effect of secondary hyperparathyroidism and hemodialysis therapy on alveolar bone and periodontium.* Journal of clinical periodontology, 2002. **29(6)**: p. 479-483.

8. Ramfjord, S.P., *The Periodontal Disease Index (PDI).* The Journal of Periodontology, 1967. **38**(6): p. 602-610.

9. Newman, M.G., et al., *Carranza's clinical periodontology*. 2011: Elsevier health sciences.

**Supplementary Table 2: Prevalence and severity of periodontitis**

| First author and year of publication | Participants | Summary findings |
| --- | --- | --- |
| Abou-Bakr. A (2022) | HD 263 | Periodontitis prevalence 85.6% |
| Borawski. J (2007) | HD 35, CAPD 33, Pre-dialysis 38, control 56 | Relatively poor periodontal health in HD group compared to others |
| Brito. F (2012) | HD 40 | Severe chronic periodontitis prevalence 55% |
| Camacho-Alonso. F (2018) | HD 120, 120 healthy controls | Significantly higher bleeding index, CPTIN, clinical attachment level, and probing depth in HD patients compared to control group, positive correlation with the duration of HD |
| Castillo. A (2007) | 104 (52 HD patients and 52 controls) | No statistically significant differences were found between the HD patients and the control group regarding bleeding index, number of teeth, or percentage of LPA > or =3 mm |
| Chen. LP (2006) | HD 253 | Periodontitis prevalence 80.6% |
| de Souza. CM (2014) | HD 122 | Chronic periodontitis Prevalence 59% |
| Dembowska.E (2022) | 200 (100 HD patients and 100 controls) | Moderate or severe periodontitis prevalence 63% |
| Kahar. P (2021) | HD 96 | Periodontal disease prevalence 86% |
| Kim. YJ (2017) | HD 115 | Moderate or severe periodontitis prevalence 47.66% |
| Kotecha.K (2022) | HD 127 | Periodontitis prevalence 69.5% |
| Lu. H (2022) | HD 63 | Moderate or severe periodontitis prevalence 88.9% |
| Marakoglu.I (2003) | HD 36, controls 36 | No significant difference in periodontal parameters (PI, GI, PD) between the two groups |
| Palmer. SC (2016) | HD 4726 | Moderate or severe periodontitis prevalence 40.6% (95% CI 38.9-42.3) |
| Parkar. SM (2012) | HD 152, control 152 | 4-5 mm pocket depth in 51.97% of HD patients and 15.13% of controls (p<0.001). No significant association between HD duration and periodontal status |
| Rahman. M.M (1992) | HD 52, KT 54, healthy control 52 | Significantly higher PI in HD patients compared to controls. No significant difference in sulcular bleeding index, pocket depth and plaque index |
| Rodrigues. VP (2014) | HD 96 | Periodontitis prevalence 59.4% |
| Ruospo. M (2017) | HD 3338 | Moderate or severe periodontitis prevalence 40.6% |
| Schmalz. G (2016) | 70 (35 HD patients and 35 KT patients) | Moderate or severe periodontal disease prevalence 57% in HD and 71% in KT |
| Schmalz. G (2016) | 217 (87 HD, 39 KT and 91 healthy controls) | Moderate or severe periodontitis prevalence 96.6% in HD and 87.2% in KT |
| Sekiguchi. RT (2012) | HD 94 | Moderate correlation between length of time on HD and DMFT index, probing depth, and clinical attachment loss. |
| Siribamrungwong. M (2012) | HD 30 | Periodontitis prevalence 63% |
| Takeuchi. Y (2007) | 81 (41 HD, 40 non-HD) | Periodontitis prevalence 71.7% |
| Tawfig. A (2016) | 120 (30 healthy, 30 Pre-dialysis, 30 HD 30 KT) | Moderate or severe periodontitis prevalence 46.6% in healthy, 70% in pre-dialysis, 83.4% in HD and 60.0% in KT |
| Veisa.G (2017) | HD 101 | Periodontitis prevalence 75.2% |
| Zhou. D (2014) | HD 612, healthy 1124 | 252 (41.2%) sextants with moderate periodontitis and 237 (38.7%) sextants with severe periodontitis in the patients undergoing Compared with 284 (23.2%) and 119 (9.7%) sextants in the control patients |

CPTIN: Community Periodontal Index of Treatment Needs, DMFT: Decayed, missing, filled teeth, GI: Gingival Index HD: Haemodialysis, KT: Kidney transplant, PD: Pocket depth, PI: Plaque Index

**Supplementary Table 3: Oral complications**

| **First author and year of publication** | **Number of participants** | **Summary findings** |
| --- | --- | --- |
| Amara Swapna. L (2013) | HD 97 | High prevalence of dry mouth (70.1%), dysgeusia (79.3%) and pocket depth >4mm (75.2%) |
| Camacho-Alonso. F (2018) | HD 120, 120 healthy controls | Significantly lower unstimulated whole saliva in HD patients compared to controls (p<0.001) |
| Cengiz. MI (2009) | HD 68, Healthy control 41 | No significant difference in DMFT between HD patients and healthy controls. Significantly higher PI, GI, PPD and LPA in HD patients compared to healthy controls (p<0.01). Significant correlation between HD duration and periodontal parameters: PI, GI, PPD and LPA |
| Gavaldá.C (1999) | HD 105, Healthy control 53 | Significantly higher plaque and calculus indices in HD patients compared to the controls (p<0.001). No significant differences in oral mucosal pathology and loss of periodontal attachment (p>0.05). Significantly higher stimulated salivary secretion in controls than HD patients (p<0.001) |
| Kopić. V (2019) | HD 40, CKD non-HD 40 | No significant difference in periodontal health between HD patients and CKD patients (stages III and IV) not on HD. |
| Limeres. J (2016) | HD 44, Healthy control 44 | Significantly higher average number of missing teeth (p=0.018), levels of supragingival bacterial plaque (p<0.001), dental calculus deposits (p<0.001) and gingival inﬂammation (p=0.01) and significantly lower saliva volume (p<0.001) in HD patients compared to health controls (p=0.018) |
| Ma.L (2016) | HD 49, PD 38, healthy controls 40 | Significantly higher gingival index and calculus surface index in HD patients compared to control (p=0.01) |
| Misaki. T (2021) | HD 80 | Significant association between the DMFT and aortic calcification indexes in HD patients. Mean DMFT was 18.5(+/-6.7) and 21.2(+/-5.6) in surviving and non-surviving HD patients after 2 year follow up. |
| Oduncuoğlu. BF (2020) | HD 63, KT 64, healthy controls 61 | Significantly higher DMFT scores and periodontal scores in HD patients than KT and control groups |
| Teratani. G (2013) | HD 98, healthy control 106 | HD patients have significantly lower number of teeth, salivary flow rate and total score of xerostomia compared to the controls |

DMFT: Decayed, missing, filled teeth, CKD: Chronic kidney disease, GI: Gingival index, HD: Haemodialysis, KT: Kidney transplant, LPA: Loss of periodontal attachment, PI: Plague index, PPD: Probing pocket depth

**Supplementary Table 4: Comorbidities and Mortality**

| **First author and year of publication** | **Number of participants** | **Summary findings** |
| --- | --- | --- |
| Camacho-Alonso. F (2018) | HD 120, 120 healthy controls | Significantly higher prevalence of depression and anxiety in HD patients than control (p < 0.001) |
| Chen. LP (2006) | HD 253 | Markers of malnutrition and inflammation are associated independently with periodontitis severity in HD patients |
| Chen. LP (2011) | HD 253 | HRs for all-cause mortality were 1.39 (95% CI, 0.83-2.34) and 1.83 (95% CI, 1.04-3.24) for moderate and severe periodontitis, respectively compared to mild/no periodontitis |
| Chen. LP (2011) | HD 253 | Prevalence of metabolic syndrome was 65.1 %among HD patients. Moderate-severe periodontitis is associated with metabolic syndrome in HD patients: OR 1.561 (95% CI, 1.121-2.166) per score of gingival inflammation severity and 1.724 (95% CI, 1.135-12.615) per score of dental plaque burden |
| Dağ. A (2010) | HD 43, Healthy 43 | Significantly higher levels of TNF-alpha and IL-8 in gingival crevicular fluid in HD patients compared to controls (p<0.001), strong, positive correlations between clinical periodontal parameters (PIGI, pocket depth) and the levels of inflammatory cytokines (TNF-alpha and IL-8) in gingival crevicular fluid from the HD patients (p<0.001) |
| Ghalwash.D.M (2022) | HD 45 | Significantly higher fibroblast growth factor 23 in GCF in patients with ESRD with periodontitis |
| Hou. Y (2017) | 136 With periodontitis 70 (51.5%) Without periodontitis 66 (48.5%) | Significantly higher levels of total cholesterol, high-sensitivity CRP, fasting blood glucose, and peripheral white blood cell counts in PD group, compared with the non-PD group (all P < .001) |
| Iwasaki. M (2018) | 211  With periodontitis 92 (43.6%) Without periodontitis 119 (56.4%) | Significant association between periodontal disease and death from pneumonia (adjusted sub hazard ratio, 3.5; 95% CI, 1.15-10.6) |
| Kopić. V (2019) | HD 40, CKD non-HD 40 | Significantly higher CRP and IL-6 levels in HD patients compared to CKD patients (stages III and IV) not on HD |
| Kovačević.P (2021) | HD 100 | 28% had sarcopenia |
| Kshirsagar. AV (2007) | HD 154 | Periodontitis was associated with low serum albumin (Adjusted OR 8.20, 95% CI 1.61 to 41.82 (p=0.01) |
| Kshirsagar. AV (2009) | HD 168 | Moderate-to-severe periodontitis in HD patients was significantly associated with death from cardiovascular causes compared with mild or no periodontal disease (HR 5.0 (95% CI, 1.2-19.1; P=0.02) |
| Lu. H (2022) | Total 138 HD 63 Healthy 75 | Significantly higher CRP, TNF-α, MCP-1, and MMP-8 levels in GCF in HD patients compared to healthy controls (p<0.05) |
| Mikami.R (2023) | HD 254 | Significant association between severe periodontitis and Malnutrition-inflammation-atherosclerosis (MIA) syndrome OR:2.64, 95% CI] 1.44-4.84 (p = 0.002) |
| Naghsh.N (2017) | HD 57 | Significantly higher Serum level of albumin (P=0.02) and ferritin (P=0.043) in patients with periodontitis compared to gingivitis |
| Pallos. D (2020) | Total 131 HD 40 CKD not on HD 91 | Significant association between HSV-1 and HCMV in HD patients |
| Rapone.B (2019) | HD 66 | Significant correlation between CRP with plaque index (p = 0.012), with bleeding on probing (p < 0.0019), and with clinical attachment level (p = 0.022) |
| Rodrigues. VP (2014) | HD 96 | Positive association of periodontitis with hypalbuminaemia (OR = 9.10, p = 0.006) and a negative association with hyperphosphatemia (OR = 0.21, p = 0.010) in HD patients |
| Ruospo. M (2017) | HD 3338 | Periodontitis is not associated with increased mortality in HD patients |
| Sharma.L (2018) | Total 100 HD 50 Healthy 50 | Significantly higher levels of CRP in HD patients compared to healthy controls (p<0.05) |
| Veisa. G (2016) | HD 200 | Association between albumin and nutritional status  with a significantly increased mortality among HD patients |

CI: Confidence Interval, CKD: Chronic kidney disease, CRP: C-Reactive Protein, ESRD: End stage renal disease, GCF: Gingival Crevicular Fluid, HD: Haemodialysis, HR: Hazard Ratio, HSV-1: Herpes Simplex Virus type 1, HCMV: Human Cytomegalovirus, IL-8: Interpeukin-8, MCP-1: Monocyte Chemoattractant Protein-1, MMP-8: Matrix Metalloproteinase-8, PD: Pocket depth, OR: Odds Ratio, TNF-alpha: Tumour Necrosis Factor-alpha,

**Supplementary Table 5: Oral health related quality of life (OHRQoL)**

| First author and year of publication | Participants | Summary findings |
| --- | --- | --- |
| Camacho-Alonso. F (2018) | HD 120, 120 healthy controls | Significantly lower OHRQoL (OHIP-14) scores in HD patients compared to healthy controls (p<0.04) |
| Dembowska.E (2022) | 200 (HD 100 and healthy controls 100) | Poor quality-of-life parameters in HD patients compared to the control group |
| Guzeldemir. E (2009) | HD 47 | Fair or poor self-perceived health in 72.7% HD patients. |
| Iwasaki.M (2017) | HD 188 | Independent relationship between severe periodontitis and decreased health related quality of life in HD patients |
| Oduncuoğlu. BF (2020) | HD 63, KT 64, healthy controls 61 | Significantly lower OHRQoL scores in HD and KT patients compared to healthy controls (p<0.05) |
| Schmalz. G (2016) | 217 (HD 87, KT 39 and healthy controls 91) | No significant difference in OHIP G14 scores between the groups |
| Veisa.G (2017) | HD 101 | Poor quality-of-life parameters in HD patients with periodontitis |

HD: Haemodialysis, KT: Kidney transplant, OHIP-14: Oral health impact profile-14, OHRQoL: Oral health related quality of life

**Supplementary Table 6: Effects of periodontal treatment**

| **First author and year of publication** | **Intervention group** | **Control group** | **Summary findings** |
| --- | --- | --- | --- |
| Chung. WC (2022) | n=7 HD patients with chronic periodontitis Non-surgical periodontal therapy | n=7 HD patients with chronic periodontitis Oral hygiene instruction | No significant difference between groups in all-cause mortality (OR 0.15, 95% CI 0.01–3.71, p = 0.244), cardiovascular events (OR 0.29, 95% CI 0.01–8.39, p = 0.470), and infection events (OR 1.88, 95% CI 0.20–17.27, p = 0.579). Significant reduction in IL-1β in GCF in the treatment group but non-significant reduction in IL-1β in serum |
| Fang. F (2015) | n=48 Non-surgical periodontal therapy and supragingival prophylaxis at the 3-month followup | n = 49 No intervention | Significant improvement in clinical periodontal parameters, interleukin-6, ferritin, albumin, creatinine, blood urea nitrogen, and transferrin levels (p<0.05) and significantly lower high-sensitivity C-reactive protein at 3 months and 6 months (p < 0.05) in intervention group compared to the control group. |
| Huang. ST (2015) | n=4451 HD patients received periodontitis treatment | n=4451 HD patients without periodontitis | Significant reduction in hospitalisation (HR 0.72 (95% CI  0.66–0.78, P < 0.001), significantly lower risk of acute and subacute infective endocarditis (HR 0.54, 95% CI   0.35–0.84, P < 0.01), pneumonia (HR  0.71, 95% CI   0.65–0.78, P < 0.001), and osteomyelitis (HR 0.77, 95% CI  0.62–0.96, P < 0.05) than the control group |
| Kadiroglu. AK (2006) | n=41 Scaling and root planning | n/a | Significant reductions in C-reactive protein levels following periodontal treatment (p < 0.001). |
| Raeesi.V (2023) | n=60 (30 HD patients with chronic periodontitis, 30 systemically healthy with chronic periodontitis) Scaling and root planning | n/a | Improvement in pocket depth (PD) and clinical attachment level (CAL) in both groups, with significantly higher mean improvement in PD in the control group compared to dialysis group |
| Vrinda.S.A (2021) | n=60 (40 HD patients with chronic periodontitis, 20 systemically healthy patients with chronic periodontitis) Scaling and root planning | n/a | Statistically significant reduction of CRP in HD patients on HD <1yr (<0.04) and in healthy controls (p=0.03), non-significant reduction in CRP in HD patients on HD for >1yr (p>0.05)) |
| Yazdi. FK (2013) | n=77 Nonsurgical periodontal treatment | n/a | Significant reductions in C-reactive protein levels (p < 0.001). |

CI: Confidence Interval, CRP: C-Reactive Protein, GCF: Gingival Crevicular Fluid, HD: Haemodialysis, HR: Hazard Ratio, PD: Pocket depth, OR: Odds Ratio
